# Genome-Wide Characterization and Transcriptomic Analysis Reveal Light-Quality-Responsive RING Zinc Finger Genes in Potatoes

**DOI:** 10.3390/plants15192915

**Published:** 2026-09-23

**Authors:** Pengbo Pan, Rong Guo, Gang Sa, Hongyu Yang, Lixiang Cheng, Jianlong Yuan, Juan Liu, Bin Yu

**Affiliations:** 1 State Key Laboratory of Aridland Crop Science, Gansu Agricultural University, Lanzhou 730070, China; 1073324020171@st.gsau.edu.cn (P.P.); chenglx@gsau.edu.cn (L.C.);; 2 College of Agronomy, Gansu Agricultural University, Lanzhou 730070, China; 3 College of Horticulture, Gansu Agricultural University, Lanzhou 730070, China; 4 College of Water Conservancy and Hydropower Engineering, Gansu Agricultural University, Lanzhou 730070, China

**Keywords:** RING zinc finger proteins, E3 ubiquitin ligase, light response, genome-wide analysis, Solanum tuberosum

## Abstract

RING-zf E3 ligases play key roles in plant development and light signaling, but their genome-wide characterization and light-responsive functions in potatoes remain largely unexplored. In this study, we identified 291 *StRING-zf* genes containing complete RING domains in the potato genome and classified them into four major RING domain types, with RING-H2 being the predominant and structurally conserved group. Evolutionary analyses indicated that gene duplication contributed to the expansion of the *StRING-zf* genes, while promoter analysis revealed abundant cis-regulatory elements associated with light, hormones, and abiotic stress responses. Transcriptome analysis of the terminal leaflets of the third leaves of potato seedlings under different light qualities, combined with qRT-PCR validation, revealed diverse and gene-specific expression patterns among *StRING-zf* genes. Five candidate genes (*Soltu.DM.01G022260.1*, *Soltu.DM.04G001050.1*, *Soltu.DM.10G023720.1*, *Soltu.DM.11G009040.1*, and *Soltu.DM.11G017440.1*) exhibited distinct light-quality-dependent expression responses. These expression patterns provide preliminary evidence for their potential involvement in light-responsive regulatory processes and ubiquitin-mediated protein modification. Overall, this study provides a comprehensive genomic and transcriptomic resource for elucidating the evolutionary characteristics and potential functions of the *StRING-zf* genes and identifies candidate genes for further functional validation of light-responsive mechanisms in potatoes.

## 1. Introduction

Ubiquitination is a highly conserved and essential post-translational modification in eukaryotes, significantly contributing to cellular homeostasis and the regulation of plant growth and development [1]. Ubiquitin is a small protein consisting of 76 amino acids (molecular weight around 8.5 kDa) and is highly conserved among eukaryotes [2]. Ubiquitination is facilitated by a series of specialized enzymes, sequentially involving the collaborative functions of ubiquitin-activating enzyme (E1), conjugating enzyme (E2), and ligating enzyme (E3). This mechanism involves the activation, transfer, and covalent attachment of ubiquitin to target proteins, referred to as the ‘three-enzyme cascade’ [3].

E3 ubiquitin ligases are important for recognizing substrates and facilitating the precise attachment of ubiquitin, hence directly influencing the specificity and regulatory pathway of ubiquitination [4]. Studies demonstrate that E3 ubiquitin ligases are crucial in plant organ development, light signal transmission, and stress responses through the selective degradation or regulation of downstream proteins [5]. For example, in Arabidopsis thaliana, DA1 collaborates with E3 ubiquitin ligases to modulate floral organ size and light signal transduction [6,7,8]; in rice, the E3 ubiquitin ligase target protein EL5 is involved in root development and drought response [9,10], whereas in pepper, the E3 ubiquitin ligase homolog CaPUB1 adversely affects desiccation and salt stress tolerance [11]. These findings jointly illustrate the pivotal function of E3 ubiquitin ligases in modulating ubiquitin-dependent mechanisms in plants. E3 ubiquitin ligases can be classified into three primary categories based on variations in their catalytic domains: HECT (Homologous to E6-AP C Terminus), U-box, and RING (Really Interesting New Gene) [12]. RING-type E3 ubiquitin ligases can directly transfer ubiquitin to substrates without establishing covalent connections with ubiquitin, constituting the most prevalent and functionally varied class in plants [13]. The RING domain often consists of 40–60 amino acids and has a distinctive Cys/His coordination motif (Cys-X_2_-Cys-X(9–39)-Cys-X(1–3)-His-X(2–3)-Cys/His-X_2_-Cys-X(4–48)-Cys-X_2_-Cys), facilitating the stable binding of two Zn^2+^ ions to create a zinc finger (zf) structure [14,15]. In contrast to other ZF structures that mainly facilitate DNA or RNA interactions, the RING motif largely engages in protein–protein interactions and the assembly of polyubiquitin complexes, demonstrating unique biological roles [16,17]. RING-zf domains can be categorized into kinds including RING-H2, RING-HC (with HCa and HCb subtypes), RING-V, RING-D, RING-S/T, RING-G, and RING-C2, based on the arrangement of Cys/His residues [18]. The RING-H2 and RING-HC types are the most conserved categories, extensively found across various plant species and have universal importance in evolutionary and functional differentiation [18].

Structural and quantitative variations among RING-zf members demonstrate significant variety across several taxa. *Arabidopsis thaliana* contains 241 RING-H2, 145 RING-HCa, and 41 RING-HCb members; *Oryza sativa* has 281 RING-H2 and 119 RING-HC members; *Malus sativa* includes 367 RING-H2, 202 RING-HCa, and six RING-HCb members; *Brassica rapa subsp. pekinensis* possesses 371 RING-H2, 215 RING-HCa, and 47 RING-HCb members, while tomato comprises 248 RING-H2, 142 RING-HCa, and 21 RING-HCb members [12,14,15,16,17,19]. In addition to differences in family size, the occurrence of less-conserved RING-zf types also varies among lineages; for example, RING-V, RING-C2, RING-S/T, and RING-G have been reported in several angiosperm species but are not universally present, whereas additional modified RING-zf types have been identified in specific lineages [18]. This indicates that the gene family has experienced significant expansion and diversification throughout plant evolution.

Various forms of RING-zf domain-mediated E3 ubiquitin ligases provide multifaceted regulatory functions in plant physiological metabolism family, hormone signaling, and stress responses. COP1 (constitutively photomorphogenic 1, RING-HC type) is a highly conserved E3 ubiquitin ligase found in almost all eukaryotic organisms [20]. COP1 often associates with SPA (suppressor of phya), facilitating the ubiquitin-mediated degradation of target proteins, including the photomorphogenesis transcription factor HY5 (long hypocotyl 5), thus regulating photomorphogenesis [21,22,23,24,25,26]. HOS1 (high expression of osmotically responsive genes 1, RING-C2 type) augments plant resilience to low-temperature stress through its interaction with ICE1, facilitating its ubiquitination and subsequent destruction [27]. NLA (nitrogen limitation adaptation, RING-HC type) is involved in the cross-regulation of nitrogen and phosphorus signaling in soybean, ensuring nutrient equilibrium [28], whereas SDIR1 (salt-and-drought-induced really interesting new gene finger1, RING-H2 type) modulates ABA signaling and responses to salt stress through the ubiquitination of its substrate SDIRIP1 (SDIR1-interacting protein1) [29]. Moreover, wheat TaRZF70-RING-H2 demonstrates tissue-specific reactions to water stress, indicating its possible function in environmental adaptation [30]. These results demonstrate that RING-zf-type E3 ubiquitin ligases play essential regulatory roles in various biological processes in plants.

Potato is a globally important food crop with extensive cultivation areas, remarkable adaptability and high yields. The quality of light is a significant environmental factor influencing potato growth and yield [31]. Plants utilize photoreceptors such as phytochrome (PHY), cryptochrome (CRY), and phototropin (PHOT) to detect light signals across various wavelengths. This regulates photomorphogenesis, photosynthesis, and metabolic activities [32,33,34]. The quality of light markedly affects plant morphological characteristics and physiological processes, with red and blue light demonstrating the most significant stimulating effects [35]. Studies have shown that white light is the main light source for potato cultivation, but different light qualities have different effects on potato plant development, which in turn affects tuber formation [36]. Blue light enhances potato growth and photosynthetic efficiency, but red light encourages chlorophyll accumulation and increases tuber weight, albeit at the expense of tuber quantity. Extended exposure to far-red and ultraviolet light may inhibit tuber development [37,38]. Previous studies have identified 77 *StRING-HC* genes in potatoes, potentially implicated in tissue formation, responses to abiotic stress, and anthocyanin synthesis [39]. Nonetheless, comprehensive studies on the potato *StRING-zf* genes are few, especially concerning their roles in light response and tuber development.

This study thoroughly identified 291 members of the *StRING-zf* genes using potato whole-genome data, analyzing their chromosomal distribution, gene structure, duplication events, evolutionary relationships, and conserved domains comprehensively. Enhanced integration with RNA-seq data examined the expression response patterns of *StRING-zf* genes under different light quality settings, identifying crucial members likely engaged in light signal transduction and tuber formation. This study establishes theoretical foundations for clarifying the functional mechanisms of the *RING-zf* genes family in the regulation of light signals and plant development in potatoes, while also serving as a reference for functional studies and molecular enhancement of *RING-zf* genes in other crops.

## 2. Materials and Methods

### 2.1. Examination and Characterization of the Potato StRING-zf Genes

We obtained the potato whole-genome sequence, transcript sequences, coding sequences, proteome sequences, and gene model annotation files in GFF3 format from the Potato Genome Sequencing Consortium (PGSC) database (https://spuddb.uga.edu/). We downloaded the hidden Markov model family (zf-C3HC4_2:PF13923, e-value < 1 × 10^−05^) and performed a genome-wide search using HMMER 3.0 with default parameters [40,41]. Concurrently, we performed NCBI BLAST+ (version 2.17.0; https://blast.ncbi.nlm.nih.gov/Blast.cgi; accessed on 21 April 2026) searches through the National Institutes of Health online (CDD version 3.21; https://www.ncbi.nlm.nih.gov/Structure/cdd/wrpsb.cgi; accessed on 21 April 2026) to evaluate potential genes utilizing both methodologies. To enhance the precision of candidate gene identification, SMART (version 10.0; https://smart.embl.de/; accessed on 21 April 2026) and NCBI were utilized to examine potato genes with RING-finger domains. Redundant sequences and proteins lacking the conserved RING-finger domain were removed, resulting in the identification of the *StRING-zf* gene family.

### 2.2. Physicochemical Assessment of the Potato StRING-zf Genes

The physicochemical characteristics of proteins encoded by the potato *StRING-zf* gene family were examined utilizing the ExPasy online platform (https://web.expasy.org/protparam/, accessed on 21 April 2026). The subcellular localization of gene family members was predicted using the online software WoLF PSORT (version 0.2; https://wolfpsort.hgc.jp/; accessed on 21 April 2026).

### 2.3. Structural Analysis of the Potato StRING-zf Genes

Alignment of *StRING-zf* gene members was conducted with the online CLUSTALW Bioinformatics Tool platform (version 2.1; https://www.genome.jp/tools-bin/clustalw; accessed on 21 April 2026) in conjunction with Jalview and MEGA software (version 11; https://www.megasoftware.net/; accessed on 21 April 2026). The *StRING-zf* genes were further categorized according to the location and quantity of metal-binding sites.

### 2.4. Chromosomal Localization, Conserved Motifs, Gene Structure, and Conserved Domain Analysis of the Potato StRING-zf Genes

Chromosomal positional data for potato was received from the PGSC database, and potato *StRING-zf* genes were annotated onto the chromosomes. Using gene annotation data (GFF3 format files) from PGSC, the software GSDS 2.0 (Gene Structure Display Server 2.0) was utilized to evaluate the family exon intron structure of potato *StRING-zf* genes. The amino acid sequences of potato *StRING-zf* genes family members were simultaneously uploaded to the web tool MEME (version 5.5.9; https://meme-suite.org/; accessed on 21 April 2026) to assess conserved motifs in the potato StRING-zf proteins, utilizing 15 motifs as the criterion. All conserved domains of the StRING-zf family proteins were examined utilizing NCBI. The outcomes of the aforementioned analyses were illustrated via TBtools.

### 2.5. Examination of Inter- and Intra-Species Collinearity Within the Potato StRING-zf Genes

Gene duplications were examined with the Multiple Collinearity Scan Toolbox (MCScanX) (version 1.0.0; https://www.rcac.purdue.edu/software/mcscanx; accessed on 22 April 2026). TBtools software (https://www.tbtools.com; accessed on 23 April 2026) was utilized to locate homologous segments among *StRING-zf* genes and to analyze collinear regions of *StRING-zf* genes across *Solanum tuberosum*, *Arabidopsis thaliana*, *Solanum lycopersicum, Nicotiana tabacum*, *Piper nigrum*, and *Oryza sativa*. TBtools was utilized to visualize the prevalence of *StRING-zf* genes duplications and their collinearity among the specified species. The ParaAT program (version 2.0; https://ngdc.cncb.ac.cn/tools/paraat?lang=zh; accessed on 23 April 2026) and KaKs Calculator software (version 2.0; https://ngdc.cncb.ac.cn/tools/kaks; accessed on 23 April 2026) were employed to compute the synonymous substitution rate (Ks), the non-synonymous substitution rate (Ka), and their ratio (Ka/Ks) [42].

### 2.6. Examination of Cis-Acting Regions Within the Potato StRING-zf Genes

Promoter sequences (2 kb upstream of the 5′ UTR) for each potato *StRING-zf* domain gene were retrieved from the potato genome and submitted to the Plant-CARE website (http://bioinformatics.psb.ugent.be/webtools/plantcare/html/; accessed on 23 April 2026) for investigation of cis-acting elements [43]. Cis-acting elements were predicted, annotated, and classified [44].

### 2.7. Plant Material, Light Quality Treatment and Determination Standard

Sterile tissue culture seedlings were propagated using the potato cultivar “Favorita”. Before transplantation, the tissue culture plantlets were subjected to a 3-day hardening-off treatment. The plants were cultivated under natural sunlight without artificial white-light supplementation with a 16 h light/8 h dark photoperiod. During the first day, the culture vessel lids were partially opened to allow gradual ventilation while maintaining relatively high humidity. During the second day, the lids were further opened to increase ventilation and gradually expose the plantlets to ex vitro conditions. On the third day, the vessels were fully opened to complete acclimatization. After hardening-off, the plantlets were carefully removed from the culture vessels, and the residual culture medium was gently washed from the roots with clean water. The plantlets were subsequently transplanted onto the cultivation substrate and maintained under the experimental conditions described above. The plantlets were then transplanted onto a sterile mixed substrate consisting of peat and vermiculite at a 1:1 ratio (pH 5.5–6.5) and grown in a family greenhouse (natural light, no white light fill). Conditions for cultivation include: Daytime temperature ranges from 20 to 25 °C, while nighttime temperature varies between 12 and 15 °C; initial relative humidity is 70% to 80%, decreasing to around 60% after hardening. Healthy leaves, free from disease and of uniform size, were cultivated for 28 days under natural light circumstances and underwent quick freezing in liquid nitrogen. These were promptly kept in a −80 °C freezer. Three plants were randomly selected as biological replicates, and the terminal leaflet of the third leaf from each plant was collected for transcriptome sequencing. These samples functioned as the primary control (CK), establishing a reference baseline to elucidate the differential impacts of natural light compared to various light quality treatments on leaf development.

Potted plants were relocated to an artificial climate chamber for diverse light quality treatments. Cultivation racks were equipped with LED (light-emitting diode) panels at the apex, facilitating adjustable illuminance. The temperature was regulated at 20 ± 2 °C photosynthetically. For the controlled-light experiments, plants were cultivated in climate chambers under a consistent 16 h light/8 h dark photoperiod with active radiation at 350 μmol/m^2^/s. Six treatment groups were created: white light (380–780 nm) (the CK represented conventional natural-light greenhouse cultivation, whereas white light represented the baseline condition for the controlled-light treatments and the white light had a spectral composition of 23.5% of red, 49.0% of green, and 27.5% of blue light), red light (625–635 nm), green light (515–525 nm), blue light (435–445 nm), ultraviolet light (390–400 nm), and total darkness (dark). The start and end times of a day are the same in both the greenhouse and the climate chamber.

Following 28 consecutive days of treatment for each group, healthy, disease-free leaves of uniform size were harvested from each treatment group in accordance with the control (CK) sample collection protocol. The leaves were swiftly frozen in liquid nitrogen and thereafter preserved at −80 °C. The chosen samples were then utilized for transcriptome sequencing. To minimize the effects of sampling time on physiological and transcriptomic responses, samples from the control (CK) and all light-treatment groups were collected at the same time and at the same developmental stage.

At the same time, tissue culture plantlets subjected to the same light quality treatments were maintained under the corresponding conditions for subsequent phenotypic and physiological measurements. The tissue culture seedlings of each light treatment were taken out after 28 days of culture to observe the growth status of test-tube seedlings, and the plant height, stem diameter, leaf number, maximum leaf area, longest root length, root number, fresh weight per plant and dry weight per plant were measured. The biology of each treatment was repeated three times and the measured data were recorded.

Two complementary experimental systems were established to characterize potato responses to different light qualities at both phenotypic and transcriptomic levels. Potted potato plants were used for transcriptome analysis, while tissue culture plantlets were used to assess growth, morphological, biomass, and physiological responses. Both experimental systems were subjected to the same light quality treatments under their respective controlled growth conditions. For transcriptome sequencing, the terminal leaflets of the third leaves were collected from potted plants at the designated sampling stage. In parallel, tissue culture plantlets exposed to the corresponding light treatments were evaluated for phenotypic and physiological traits. The transcriptomic and phenotypic analyses were therefore performed using independent experimental materials and were integrated to identify and characterize candidate *StRING-zf* genes potentially involved in light-responsive regulation.

### 2.8. Transcriptome Sequencing and Bioinformatic Analysis

Library preparation and sequencing, specifically targeting mRNA-seq for comprehensive mRNA sequencing utilizing eukaryotic reference-based transcriptome methodologies, were conducted by Shanghai Ouyi Biomedical Technology Co., Ltd. Raw sequencing data in fastq format were produced using high-throughput sequencing. Raw reads were subjected to quality filtering to yield high-quality readings appropriate for future processing. Preliminary quality control and adapter elimination were conducted via Trimmomatic [45]. Consequently, low-quality bases and N-bases were eliminated, resulting in high-quality clean reads. Hisat2 [46] was utilized to align the clean reads to the potato reference genome, employing default program parameters. The quality of the sample was evaluated via genomic alignment coverage. The alignment outcomes between clean readings and the potato reference genome were preserved as binary files (BAM files). Gene FPKM [47] expression values were quantified via the Cufflinks [48] program. The quantification of gene expression differences was achieved by obtaining the read counts mapping to each gene across samples utilizing HTSeqCount [49]. FPKM > 10 is often used as a practical threshold for screening reliable expressed genes [50]. Data were normalized utilizing the estimate Size Factors function from the DESeq R package (version 1.8.3) [51], and the nbinom test function was applied to compute *p*-values and fold change values for differential analyses. Genes with differential expressions were identified using *p*-values < 0.05 and fold changes > 2. The genes were subjected to GO enrichment analysis to determine the principal biological activities or pathways influenced [52,53]. The target gene set was compared with the preset GO term database by ORA (https://biit.cs.ut.ee/gprofiler/gost; accessed on 21 April 2026) analysis, and the statistical test was used to determine whether the number of genes in a GO term was significantly more than random expectations. Expression patterns among samples were illustrated by heatmaps.

### 2.9. Expression Study of the StRING-zf Genes Under Different Light Qualities and qRT-PCR Analysis

Operating according to the manufacturer’s instructions, Trizol reagent (Invitrogen) was used to extract total RNA from the third expanded leaf of 28 d tissue culture seedlings under different light qualities. RNA integrity and quality were detected by NanoDrop 1000 and electrophoresis. First-strand cDNA was synthesized by the SuperScript II Reverse Transcriptase (Invitrogen). qRT-PCR research revealed 5 candidate genes under different light qualities. Table S1 in Supplementary File S7 lists the qRT-PCR primers. The qRT-PCR was done using the QuantiFast^®^ SYBR^®^ Green PCR kit (QIAGEN GmbH, Hilden, Germany) according to the manufacturer’s instructions, with three replications for each sample. Relative gene expression levels were calculated using the 2^−ΔΔCt^ method, where ΔCt = Ct_target − Ct_reference and ΔΔCt = ΔCt_sample − ΔCt_control. Actin was used as the reference gene [19].

## 3. Results

### 3.1. Comprehensive Analysis of Potato StRING-zf Genes Across the Genome

A total of 306 candidate genes were initially identified, and 291 genes containing complete RING domains with eight conserved metal-binding residues were retained for further analysis. A thorough examination of *StRING-zf* genes included gene ID, chromosomal start and end positions, protein length, molecular weight, theoretical isoelectric point, subcellular localization, RING domain type, and RING domain length (Supplementary File S1: Table S1). *StRING-zf* genes varied in length from 96 (*Soltu.DM.05G013050.1*) to 1327 (*Soltu.DM.03G002260.1*) amino acids, with molecular weights spanning from 10.70814 kDa (*Soltu.DM.05G013050.1*). The broad variation in protein size and pI suggests considerable structural and physicochemical diversification among the *StRING-zf* proteins, potentially reflecting differences in domain organization and functional specialization with 145.81891 kDa (*Soltu.DM.03G002260.1*) and isoelectric points (pI) ranging from 3.86 (*Soltu.DM.02G000800.1*) to 10.31 (*Soltu.DM.06G015810.1*). Subcellular localization predictions indicated that 132 StRING-zf proteins are localized to the nucleus, 77 to chloroplasts, one to both the Golgi apparatus and plasma membrane, and one to both the cytoplasm and nucleus. These two genes are the only genes identified in the two organelles, suggesting that they may have unique biological roles and therefore warrant further investigation (Figure 1). Collectively, the marked diversity in protein properties and subcellular distribution indicates that the StRING-zf family is structurally and functionally heterogeneous rather than representing a uniform group of ubiquitin ligases.

The chromosomal distribution of potato *StRING-zf* members was investigated using genome annotation files, illustrating the uneven distribution of the 291 discovered *StRING-zf* genes across the 12 chromosomes. Chromosome 2 contains the greatest number of genes, comprising 42 *StRING-zf* genes, while chromosomes 8 and 9 possess the least, with 18 and 14 genes, respectively. The bulk of *StRING-zf* members are predominantly located at the terminal regions of chromosomes, specifically chromosomes 1, 4, 11, and 12. Analysis of gene density files indicated that *StRING-zf* genes primarily localize at the ends of chromosomes, exhibiting a dense distribution in these terminal areas, which implies structural conservation and gene clustering (Figure 2).

A collinearity study was performed to further examine the duplication events of *StRING-zf* genes, illustrating that 85 pairs of duplicated genes were identified among the 291 *StRING-zf* genes. (Figure 3). The duplication genes of *StRING-zf* genes were primarily located on chromosome 1 (15 pairs), chromosome 2 (25 pairs), chromosome 3 (10 pairs), and chromosome 4 (13 pairs). Among the 85 identified gene duplication events, 77 (90.59%) were segmental duplications, whereas eight (9.41%) were tandem duplications. These results indicate that segmental duplication was the predominant duplication mode contributing to the expansion of this gene family, whereas tandem duplication occurred at a relatively low frequency. Therefore, segmental duplication may have played a more important role in the expansion and evolution of this gene family. Tandem duplications were identified on chromosome 2, indicating that these duplicated genes may retain similar functions or undergo functional divergence during evolution. To further examine the evolutionary selection forces on the *StRING-zf* genes, the non-synonymous substitutions (Ka), synonymous substitutions (Ks), and Ka/Ks ratios were computed for RING gene pairs. All duplicate genes displayed Ka/Ks ratios under one; the KS/KA ratios ranged from 0.653 to 0.069, indicating that these duplicated genes may have undergone strong purifying selection (Supplementary File S2, Table S1), signifying that this family is predominantly influenced by purifying selection. Furthermore, consistent GC content was noted across chromosomes, signifying conserved gene function and structure for *StRING-zf* genes.

### 3.2. Identification of RING Domains in the StRING-zf Proteins

According to the types of amino acid residues at eight metal-ligand binding sites and the inter-residue distances, numbers 1–8 indicate the eight conserved metal-binding residues of the RING domain. Zn^2+^ represents the zinc ion coordinated by these conserved residues (Table 1).

The 291 putative RING domains were classified into four major types: RING-H2 (230, 79.04%), RING-HCa (57, 19.59%), RING-HCb (2, 0.69%), and RING-V (2, 0.69%) (Supplementary File S1, Table S2). RING-H2 was the predominant type, accounting for nearly four-fifths of the identified RING domains, whereas the RING-HC subtypes collectively comprised 59 members (20.27%) (Figure 4). The detailed classification further showed that RING-HC domains could be divided into RING-HCa and RING-HCb according to the distance between the metal-ligand residues ml7 and ml8, with 57 and two members, respectively (Figure 5). In addition to these canonical RING types, two members were classified as the atypical RING-V type (Figure 6).

The RING-V domain is characterized by an inversion of the conserved metal-ligand residues, with a Cys residue at ml4 and a His residue at ml5, distinguishing it from the corresponding residue arrangements in conventional RING domains. Notably, the strong predominance of RING-H2 and RING-HC domains indicates that canonical RING configurations constitute the major structural forms within the StRING-zf gene family, whereas the rare occurrence of RING-V suggests that atypical RING configurations represent a minor evolutionary diversification of the family. Given that the RING domain serves as the core structural module of many E3 ubiquitin ligases, variation in metal-ligand residue composition and spacing may affect domain conformation and potentially contribute to functional diversification among StRING-zf proteins.

### 3.3. Phylogenetic, Motif, and Structural Study of StRING-zf Genes

The 291 identified StRING-zf proteins were analyzed to enhance comprehension of their structural diversity. Through the enhanced alignment of the genomic DNA sequences of *StRING-zf* genes, the annotation of its exons, introns, UTR structures, and motif features was conducted. Figure 7 shows only phylogenetic trees for RING-HCa, RING-HCb, and RING-v, while the phylogenetic tree for RING-H2 will be provided as a supplementary figure in the Supplementary Material (Supplementary File S1, Table S2).

An unrooted phylogenetic tree based on the conserved domains of 291 StRING-zf proteins was built. The amino acid sequences of the StRING-zf proteins were aligned using MUSCLE in MEGA 11 (https://www.megasoftware.net/; accessed on 21 April 2026). A maximum-likelihood (ML) phylogenetic tree was constructed based on the best-fit amino acid substitution model determined by the model-selection analysis in MEGA 11. Node reliability was evaluated using 1000 bootstrap replicates. The resulting tree was exported in Newick format and subsequently visualized and annotated using iTOL. The figure illustrates that RING-H2 and RING-HCa types demonstrate unique clustering effects. The figure demonstrates a clear clustering effect for the RING-H2 and RING-HCa subtypes, showing relatively clear phylogenetic clustering patterns (Supplementary Figure S1a and Figure 7a). In contrast, the RING-HCb and RING-V subtypes were distributed more sporadically across the phylogenetic tree, without forming distinct evolutionary clusters (Figure 7a). The analysis of StRING-zf proteins motif identified 15 conserved motifs, which were used to create a schematic representation of StRING-zf proteins structures (Supplementary Figure S1b and Figure 7b). The RING-H2 type primarily consists of Motifs 2, 1, and 3; the RING-HCa type predominantly features Motifs 7, 1, and 3. The RING-V type includes Motifs 5, 2, 1, and 3, whereas the two protein structures of the RING-HCb type encompass unique motif combinations. Two types are Motifs 1, 4, 6, 10, and 13 (*Soltu.DM.03G002260.1*) and Motifs 1, 3, 7, and 13 (*Soltu.DM.04G034740.1*) (Supplementary Figure S1b and Figure 7b).

Fifty-four unique protein domain families were identified in the StRING-zf proteins. In addition to the essential RING-zf domain, the domain families found in several RING-zf subtypes exhibited considerable variation. The RING-H2 type largely includes the HRD1 superfamily (E3 ubiquitin ligases involved in ER-associated protein degradation and protein quality control) (19 times, 6.53%), WAK-binding superfamily (cell wall signaling and plant growth and development) (6 times, 2.62%) and COG5540 superfamily (diverse cellular and metabolic processes) (25 times, 8.59%) The RING-HCa type predominantly comprises the PLN03208 superfamily (diverse cellular and metabolic functions in plants) (15 times, 5.15%) and HepA superfamily (transcription regulation and RNA polymerase recycling) (7 times, 2.41%). The RING-HCb and RING-V types possess no shared domain families aside from the RING-zf domain (Supplementary Figure S1d and Figure 7d). The number of exons in the 291 *StRING-zf* genes ran specific from one to 21, with a mean of 5.21 and a median of four exons. Genes containing three exons were the most prevalent (89 genes, 30.6%), followed by genes with four exons (36 genes, 12.4%) and one exon (46 genes, 15.8%). Specific *StRING-zf* genes possess exceptionally lengthy introns, including *Soltu.DM.03G029850.1* and *Soltu.DM.06G015810.1* (Supplementary Figure S1c and Figure 6c).

### 3.4. Interspecies Homology Study of the Solanum tuberosum L. RING Zinc Finger Genes

An interspecies synteny analysis was performed on potato and five representative plant species to further examine phylogenetic history of the *StRING-zf* genes, comprising four dicotyledons (*Arabidopsis thaliana*, *Solanum Lycopersicon*, *Capsicum annuum*, and *Nicotiana tabacum*) and one monocotyledon (*Oryza sativa*) (Figure 8). A total of 193, 212, 47, and 42 collinear gene pairs were found in *Arabidopsis thaliana, Solanum lycopersicon, Capsicum annuum, and Nicotiana tabacum*, respectively, while 57 collinear gene pairs were detected in rice. The calculation of Ka/Ks ratios for potato and rice indicated that the majority of collinear gene pairs were NAN (52 pairs of NAN, 91.23%), signifying that most mutation sites were synonymous mutations. This indicates significant sequence divergence, vast evolutionary distance, and remote phylogenetic relationships. The average Ka/Ks ratios for potato in comparison to four dicotyledonous plants were 0.19 (124 effective pairs), 0.30 (212 effective pairs), 0.28 (45 effective pairs), and 0.27(40 effective pair), respectively (Table 2). All valid collinear gene pairs included in the mean-value calculations exhibited Ka/Ks ratios below one, suggesting that the RING-zf gene family has predominantly undergone purifying selection during evolution. The relatively high proportion of NAN pairs also indicates that sequence divergence in some collinear gene pairs may have been too extensive for reliable Ka/Ks estimation.

### 3.5. Examination of Cis-Acting Regions Within the StRING-zf Genes Promoter

In-depth examination of cis-acting regions in the promoters of *StRING-zf* genes is essential for understanding the regulation of these genes. An examination of the 2000 bp upstream region of the *StRING-zf* genes identified 66 categories (6204 elements) of cis-acting elements inside the promoter region of 291 *StRING-zf* genes. These elements are correlated with undetermined parameters related to light response, stress/growth-related components, and hormonal response (Figure 9b).

Within the *StRING-zf* genes, 37 categories of light-responsive elements were identified, primarily consisting of 654 (23.6%) Box 4, 546 (19.7%) G-box, 269 (9.8%) GT1-motif, 233 (8.4%) TCT-motif, and 173 (6.2%) GATA-motif elements (Figure 9a). Of the 66 types of cis-acting elements, the light-responsive regulatory elements Box 4 and G-box were the most prevalent, suggesting a strong association of the *StRING-zf* genes with light responsiveness and phototransduction. Within the *StRING-zf* genes, *Soltu.DM.08G012290.1* and *Soltu.DM.02G025140.1* exhibit the highest quantities of Box 4 and G-box regulatory elements, respectively, indicating that these two genes are involved in light signal response. Hormone-related elements consisted of 18 categories, mostly including 371 TGACG-motif elements 30.3% of hormone-related elements and 366 CGTCA-motif elements responsive to methyl jasmonate, followed by 436 ABREs (17.9%) responsive to abscisic acid (Figure 9a). Of the 11 stress-related elements, 525 anaerobic induction factors comprised 52.4% of the total stress-related elements. This suggests that the *StRING-zf* genes may be involved in the metabolic regulation of plant responses to hypoxic stress (Figure 9a). The quantities and categories of the other cis-acting elements are specified in the tables (Supplementary File S3: Tables S1 and S2).

### 3.6. The Effect of Different Light Qualities on the Growth of Potato Tissue-Cultured Seedlings

The promoter analysis revealed that a large number of light-responsive cis-acting elements were present in the promoters of *StRING-zf* genes, suggesting that members of this gene family may participate in light-responsive regulatory processes. To further investigate whether different light qualities were associated with phenotypic changes in potato tissue-cultured seedlings, the effects of different light treatments on plant growth and biomass accumulation were subsequently evaluated.

After 28 days of treatment, significant differences were observed among light treatments in several growth-related traits, including plant height, stem diameter, leaf number, maximum leaf area, root length, root number, fresh weight, and dry weight (Table 3 and Table 4). The data are presented as means ± standard deviations, and different lowercase letters indicate significant differences at *p* < 0.05. Blue light resulted in the greatest stem diameter (1.78 ± 0.39 mm) and leaf number (16 ± 2.02), although plant height under blue light (8.66 ± 0.71 cm) was lower than that under green and dark treatments. Red light promoted root elongation, with the longest root length reaching 9.89 ± 1.12 cm, which was significantly higher than that observed under the other light treatments. White light resulted in relatively high biomass accumulation, with fresh weight and total dry weight reaching 0.66 ± 0.04 g and 0.16 ± 0.01 g per plant, respectively. In contrast, green and dark treatments resulted in greater plant height (14.55 ± 1.00 cm and 14.71 ± 1.28 cm, respectively), but were associated with lower stem diameter, leaf number, root development, and biomass accumulation. This combination of increased plant height and reduced biomass-related traits suggests an elongated growth phenotype under these conditions. Ultraviolet treatment substantially reduced several growth-related traits, particularly plant height, maximum leaf area, root length, and dry weight. (Figure 10).

Taken together, the phenotypic results demonstrated that light quality strongly affected the morphological development and biomass accumulation of potato tissue-cultured seedlings, with distinct light qualities preferentially affecting different growth traits. To further determine whether the observed phenotypic responses were associated with changes in the transcriptional activity of *StRING-zf* genes, transcriptome data were subsequently analyzed to characterize their expression profiles under different light treatments.

### 3.7. Identification of the Expression Profiles of StRING-zf Genes and qRT-PCR Analysis

To further investigate whether the light-responsive cis-acting elements identified in the promoters of *StRING-zf* genes were associated with changes in gene expression, RNA-seq data were used to characterize the expression profiles of *StRING-zf* genes under different light treatments.

Among the 291-candidate *StRING-zf* genes, 193 genes with detectable RNA-seq signals were retained for subsequent expression analysis. In this analysis, genes with an FPKM value >10 were defined as highly expressed, whereas genes with lower expression levels were considered lowly expressed. Genes without detectable expression were excluded from comparisons of expression patterns. This criterion was applied consistently to all light treatments and the control (CK) (Supplementary File S4, Tables S1 and S2). The majority of *StRING-zf* genes within the RING-H2 category demonstrated minimal expression levels across various light treatments, with several genes exhibiting negligible expression under all light conditions. Examples comprise *Soltu.DM.01G043210.1*, *Soltu.DM.02G009750.1*, *Soltu.DM.10G005870.1* and *Soltu.DM.11G009060.1*. In contrast, most *StRING-zf* genes within the RING-HCa group demonstrated substantial expression under all light treatments, as illustrated by *Soltu.DM.10G004230.1* and *Soltu.DM.10G026870.1*. The *StRING-zf* genes exhibited expression in both the RING-HCb and RING-V groups (Figure 11). These results indicate that the transcriptional activity of *StRING-zf* genes is heterogeneous among subgroups and may be differentially associated with light conditions.

To identify genes showing treatment-associated expression patterns, genes with FPKM >10 were further compared between each light treatment and CK. The Venn diagram analysis revealed that most highly expressed genes were shared between CK and the light-treatment groups, with the overlap generally exceeding 90%, whereas only a small number of highly expressed genes were unique to individual light treatments (Figure 12). Specifically, four genes were uniquely highly expressed in the blue-light treatment, two in the dark treatment, four in the green light treatment, three in the red light treatment, two in the UVA treatment, and two in the white-light treatment (Supplementary File S5, Table S1). The treatment-specific expression patterns further indicated that certain *StRING-zf* genes may respond preferentially to particular light conditions. For example, *Soltu.DM.11G009040.1* was identified among the genes specifically highly expressed under blue light, whereas *Soltu.DM.01G022260.1*, *Soltu.DM.04G001050.1*, and *Soltu.DM.10G023720.1* occurred in multiple treatment-specific sets, particularly those associated with red and green light. These patterns suggest that individual *StRING-zf* genes may exhibit distinct transcriptional responses to different light qualities rather than responding uniformly to light treatment.

An UpSet analysis was subsequently performed to examine genes shared among the highly expressed gene sets of CK and the six light treatments. A total of 133 genes were highly expressed across all examined conditions (Figure 13), indicating that these genes maintained relatively stable transcriptional activity under the tested light regimes. Rather than being considered completely insensitive to light, their stable expression suggests that they may perform basal cellular functions that are maintained across different light environments. In contrast, four genes, including *Soltu.DM.01G029970.1*, *Soltu.DM.03G003460.1*, *Soltu.DM.10G025570.1*, and *Soltu.DM.10G025580.1*, showed high expression in CK but did not meet the high-expression criterion in the examined light-treatment groups. In addition, *Soltu.DM.11G009040.1* met the high-expression criterion specifically under blue light but not under the other tested conditions (Figure 13). These treatment-associated patterns provide candidate genes for further investigation of light-quality-dependent regulation. The expression results were consistent with the promoter analysis described above. The identification of abundant light-responsive cis-acting elements in the promoters of StRING-zf genes, together with the treatment-dependent expression patterns observed in the RNA-seq data, provides complementary evidence that members of the *StRING-zf* genes may participate in light-responsive regulatory networks. However, the presence of cis-acting elements alone does not establish direct transcriptional regulation; therefore, the observed association between promoter composition and expression patterns should be considered as predictive evidence requiring further experimental validation.

Subsequent GO enrichment analysis of these treatment-specific highly expressed genes indicated their primary enrichment in molecular functions and biological processes, including ‘metal ion binding’, ‘ubiquitin protein ligase activity’, and ‘proteasome-mediated ubiquitin-dependent protein catabolic process’. This indicates that light treatments may affect cellular physiology by modulating processes such as protein breakdown and metal ion binding. Additionally, certain genes with high expression levels were linked to stress-response-related gene ontology categories, including “response to water deprivation” and “response to chitin”. (Supplementary File S6, Table S1). This indicates that differing light conditions affect basal metabolism and may potentially trigger adaptive responses to environmental challenges in plants (Figure 14).

Integrated analysis of the Venn diagram and UpSet plot identified five differentially expressed genes, *Soltu.DM.01G022260.1* (RING-H2)*, Soltu.DM.04G001050.1* (RING-H2), *Soltu.DM.10G023720.1* (RING-H2)*, Soltu.DM.11G009040.1* (RING-H2), and *Soltu.DM.11G017440.1* (RING-Hca), with FPKM values exceeding 10 under multiple light quality treatments. The relatively high expression of these genes across different light conditions indicates their potential involvement in light-quality responses in potato, making them promising candidates for further functional characterization.

Different light quality treatments significantly affected the expression levels of *StRING-zf* genes, and there were significant differences in the responses of different genes to light quality. Among them, *Soltu.DM.01G022260.1* gene had the highest expression level under red light treatment, followed by green light and ultraviolet light treatment. The *Soltu.DM.04G001050.1* gene also reached the maximum expression level under red light, while the expression level was relatively low under white light and dark treatment. *Soltu.DM.10G023720.1* gene responded most obviously to red light. Red light treatment significantly increased its expression level, while ultraviolet light and dark treatment also showed higher expression; the expression level of *Soltu.DM.11G009040.1* gene reached the peak under blue light conditions, which was significantly higher than that under other light quality treatments. The *Soltu.DM.11G017440.1* gene showed a green-light-induced expression pattern, and the expression level was the highest under green light treatment (Figure 15).

## 4. Discussion

### 4.1. Systematic Identification, Evolutionary Characteristics, and Subcellular Localization of the StRING-zf Genes

This research methodically identified the *StRING-zf* genes throughout the complete genome of potato (*Solanum tuberosum* L.). The RING-zf domain has been thoroughly investigated in several plants, including Arabidopsis, rice, and poplar [12,17,19,54,55,56]; however, a systematic investigation is still absent for potato. Genome-wide screening yielded 306 candidate genes, of which 291 possessed intact RING domains and were chosen for further detailed analysis. Analysis indicated an irregular distribution of these genes over 12 chromosomes, with chromosome 2 containing the highest quantity. Many genes are concentrated near the telomeric ends of chromosomes, indicating that these regions are highly conserved throughout species while also displaying increased rates of sequence change, which may enable swift adaptations to selective pressures. Subsequent examination of synteny and duplication events indicated that the extension of the *StRING-zf* genes was predominantly influenced by intrachromosomal segment duplication and a limited number of tandem duplications, especially localized on chromosomes 2 and 4. This illustrates active genetic recombination and functional preservation.

The computation of the ratio of non-synonymous (Ka) to synonymous (Ks) substitutions demonstrated Ka/Ks values constantly under one, signifying robust purifying selection throughout the family’s history and a propensity for protein functional stability. These findings demonstrate an evolutionary trend in the *StRING-zf* genes in potato, marked by simultaneous specialized expansion and functional preservation. A subcellular localization study reveals that most StRING-zf proteins are situated in the nucleus, implying a fundamental role in regulating gene expression via facilitating transcriptional control and signal transmission. A subset of proteins localizes to chloroplasts, the principal site of photosynthesis, suggesting potential roles in chloroplast formation, photosynthetic control, and energy metabolism. The proteins Soltu.DM.01G029660.1 and Soltu.DM.01G029670.1 are anticipated to be exclusively localized to peroxisomes. Peroxisomes are essential for plant growth, development, and responses to diverse environmental challenges. Their main roles include fatty acid breakdown, plant hormone production, the glyoxylate cycle, photorespiration, and detoxification of reactive oxygen species [57]. StRING-zf proteins demonstrate varied intracellular distribution patterns, indicating multifaceted regulatory functions in cellular differentiation and environmental adaptability.

### 4.2. Classification of Structural Domain Attributes of StRING-zf Proteins

Analysis of RING domain types reveals that *StRING-zf* genes is predominantly classified into two principal categories: RING-H2 and RING-HC, with RING-H2 being the most prevalent, constituting 79.04%, a trait common in dicotyledonous plants like Arabidopsis, tomato, rapeseed, and apple [12,15,17,19], highlighting the prevalence and functional importance of this subtype in plant evolution. The RING-HC category can be further classified into the RING-HCa and RING-HCb subtypes. A limited quantity of atypical RING-v kinds was identified, displaying a metal ligand site configuration to that of conventional RING-HC, indicating they may have unique ubiquitin ligase activities or substrate recognition specificities. Significantly, the RING-C2, RING-S/T, RING-G, and RING-D types previously identified in Arabidopsis were absent in potato, indicating a possible domain-specific loss or functional transformation during the evolution of potato. Additionally, 13 proteins were designated as ‘incomplete’ RING domains owing to the lack of one or more metal ligands (Supplementary File S1: Table S3), which may influence their functionality. These structural changes collectively signify the functional and evolutionary diversification of StRING-zf proteins.

### 4.3. Characteristics of RING Domain Combinations and Potential Functional Divergence

An exhaustive examination of phylogeny, motifs, and domains uncovers notable disparities in motif composition and domain combinations across StRING-zf proteins of various RING types, indicating possible functional distinctions. This work uncovered 54 additional domain families outside the conventional RING-zf domain, about twice the number found in Arabidopsis [12], illustrating the structural intricacy of the *StRING-zf* genes. RING-H2-type proteins often contain domains like HRD1 and WAK bind, indicating a possible role in ubiquitin-mediated protein degradation and cell wall signaling pathways [58,59]. RING-HCa-type proteins are abundant in PLN03208 and HepA domains, likely playing a role in plant-specific physiological and metabolic activities [60]. Furthermore, specific domains, including the zinc_ribbon_9 and zf-CHY families, are linked to metal ion binding [61]. The Brcat_Rcat_RBR family, which encompasses the RBR domain, may modulate ubiquitination activity via an autoinhibitory mechanism [62]. The GUB_WAK_bind and WAK_assoc families, as cell-wall-associated kinase domains, presumably play vital roles in signal transmission between the cell wall and cytoplasm, as well as in stress responses [63,64]. The lack of shared accessory domains in RING-HCb and RING-V suggests that these two RING-zf subtypes may be more functionally differentiated, although experimental evidence is required to establish a direct relationship between domain divergence and functional specialization. These domains demonstrate significant conservation in plants like tomato and Chinese cabbage, suggesting their functional universality and stability across plant evolution [12,15,17,55].

### 4.4. Characteristics of Cis-Acting Elements and Putative Regulatory Networks of the StRING-zf Genes Promoter

Analysis of cis-acting elements reveals that the promoter region of the *StRING-zf* genes is abundant in regulatory elements linked to light response, hormonal response, and abiotic stress. Light response elements are predominantly found in Box 4 and G-box motifs, indicating a strong association of this family with light signaling pathways. Box 4 is acknowledged for its role in floral organ development, light, jasmonic acid, and abscisic acid signaling [65]. The G-box, a crucial cis-acting element [66], facilitates various induction routes, including light signaling, jasmonic acid, and abscisic acid [65]. The significant abundance of light-responsive elements indicates that *StRING-zf* genes may be involved in light-induced ubiquitin-dependent regulatory networks, therefore playing a crucial role in phototransduction and photomorphogenesis.

Moreover, the extensive presence of hormone-related components further elucidates the probable connection between *StRING-zf* genes and plant hormone regulation networks. The prevalence of methyl jasmonate response elements (TGACG-motif, CGTCA-motif) and abscisic acid response elements (ABREs) indicates that this gene may participate in transcriptional regulation within the jasmonic acid and ABA signaling pathways, influencing plant growth, development, and stress responses. The elevated prevalence of ABRE components among abiotic-stress-related factors suggests their possible involvement in responses to drought, excessive salinity, and other detrimental situations [63]. ARE elements, intimately linked to auxin-responsive gene promoters, are identified by ARF transcription factors to modulate plant growth and development [67]. The systematic enhancement of these elements suggests that the *StRING-zf* genes have significant regulatory potential in the integration of light signals, hormonal signals, and abiotic stress response pathways. It may serve a crucial function in light-induced ubiquitination regulatory networks, perhaps offering a genetic foundation for potato adaptability in intricate settings.

### 4.5. Distinct Expression Profiles of the StRING-zf Genes Under Varying Light Quality Treatments

The response of potato plant development to different light qualities was significantly different. Gene expression profiling demonstrated that the *StRING-zf* genes show considerable sensitivity to light treatments. Various light quality treatments (blue light, red light, green light, UVA, etc.) significantly affected the expression of this gene family. Among these, RING-HCa-type members exhibited notably vigorous expression responses, while the majority of RING-H2-type genes displayed diminished expression levels across all light quality situations. Among the 193 expressed *StRING-zf* genes, five genes with relatively high expression levels (FPKM > 10) across the different light-spectrum treatments were selected as candidate light-spectrum-responsive genes. These genes included *Soltu.DM.01G022260.1*, *Soltu.DM.04G001050.1*, *Soltu.DM.10G023720.1*, *Soltu.DM.11G009040.1*, and *Soltu.DM.11G017440.1*. The first four genes were classified as RING-H2, whereas *Soltu.DM.11G017440.1* belonged to the RING-HCa subtype. Domain analysis showed that no additional conserved domain families were detected in the first four proteins, while *Soltu.DM.11G017440.1* contained an SPX superfamily domain in addition to the RING-HCa domain. The RING domain is characteristic of RING-type E3 ubiquitin ligases and is associated with ubiquitination-mediated protein regulation. The presence of an additional SPX domain in *Soltu.DM.11G017440.1* may provide an additional functional module related to phosphate and nutrient signaling, potentially contributing to the integration of environmental and nutritional signals. These structural differences suggest that the five-candidate StRING-zf proteins may have distinct functional properties in regulating plant responses to different light spectra. Among them, red light and blue light significantly promoted the expression of most genes, while the expression levels of related genes under white-light and dark treatment were relatively low, indicating that the difference in light quality may affect the growth and development of potato and the mechanism of light signal response by regulating the transcription level of specific genes.

Subsequent investigation indicated that the promoter regions of these essential genes encompass characteristic light response elements, including Box 4 and G-box. Their subcellular localization includes the nucleus, chloroplasts, and other organelles, indicating they may facilitate light responses via transcriptional control or protein modification pathways. GO enrichment analysis reveals that these light-responsive genes predominantly engage in biological processes like ‘ubiquitin ligase activity’, ‘proteasome-mediated ubiquitin-dependent protein degradation’, and ‘metal ion binding’. This indicates that *StRING-zf* genes may facilitate light signal transduction by modulating protein stability. Furthermore, specific genes are associated with stress-related mechanisms, including ‘water shortage response’ and ‘chitin response’, suggesting intricate cross-regulatory interactions between light signaling networks and stress response pathways. This corresponds with the functional attributes of RING-type E3 ligases, which are extensively engaged in signal integration during plant stress responses [68].

Prior research demonstrates that plant light signals can affect the stability and functionality of photoreceptors and subsequent transcription factors (including the HY5 and COP1 pathways) through the modulation of E3 ubiquitin ligase-mediated protein degradation [69]. Based on these findings, it is hypothesized that the *StRING-zf* genes may be involved in a comparable light-dependent protein degradation regulation network. It establishes a dynamic equilibrium between photomorphogenesis and environmental adaptation by altering the homeostasis of photoreceptors and downstream transcriptional regulators. This result offers fresh theoretical support for clarifying the ubiquitin-dependent light response mechanism in potato.

### 4.6. Prospective Developments

This study thoroughly clarifies the composition, structural attributes, and light-responsive expression patterns of the potato *StRING-zf* genes, providing a molecular basis for further exploration of its function in light signal transduction and stress responses. Subsequent research should utilize functional validation techniques like CRISPR/Cas9 or VIGS to verify the biological roles of essential *StRING-zf* genes. In addition to the effects of individual wavelengths, the relative proportion of red and blue light is an important factor influencing potato growth and development under controlled-environment conditions. Different red-to-blue (R) ratios can modify plant morphology, photosynthetic performance, biomass accumulation, and tuber development, indicating that the effects of light quality depend not only on the presence of specific wavelengths but also on their relative proportions. This aspect is particularly relevant to greenhouse production of virus-free potato seed tubers, where LED lighting is increasingly used to provide supplemental or controlled illumination [70]. Therefore, optimizing the R ratio may provide an effective strategy for regulating potato growth and improving the efficiency of greenhouse seed-tuber production. Future studies should further evaluate the responses of potato plants to different combinations and ratios of red and blue light to identify spectral regimes suitable for controlled-environment cultivation [70].

## 5. Conclusions

This study systematically identified the *StRING-zf* genes at the whole-genome level in potatoes, revealing 291 members and clarifying their structural properties and evolutionary tendencies. The family can be categorized into four subclasses, with the RING-H2 type being the main category demonstrating significant structural conservation. A promoter study reveals that the majority of *StRING-zf* genes possess cis-regulatory elements linked to light response, hormone response, and stress response, indicating their possible participation in various regulatory signaling. Expression profiling indicates that *StRING-zf* genes demonstrate markedly distinct transcriptional responses to light quality treatments, with *Soltu.DM.01G022260.1, Soltu.DM.04G001050.1, Soltu.DM.10G023720.1, Soltu.DM.11G009040.1*, and *Soltu.DM.11G017440.1* being significantly upregulated under specific light quality conditions. It is hypothesized that these genes may be involved in the regulation of photomorphogenesis via E3 ubiquitin ligase-mediated protein degradation pathways. This study revealed the structural characteristics and sensitivity to light quality of the *StRING-zf* genes, and provided an important reference for molecular design and functional gene mining of potato varieties with high photosynthetic efficiency.

## Figures and Tables

**Figure 1:**
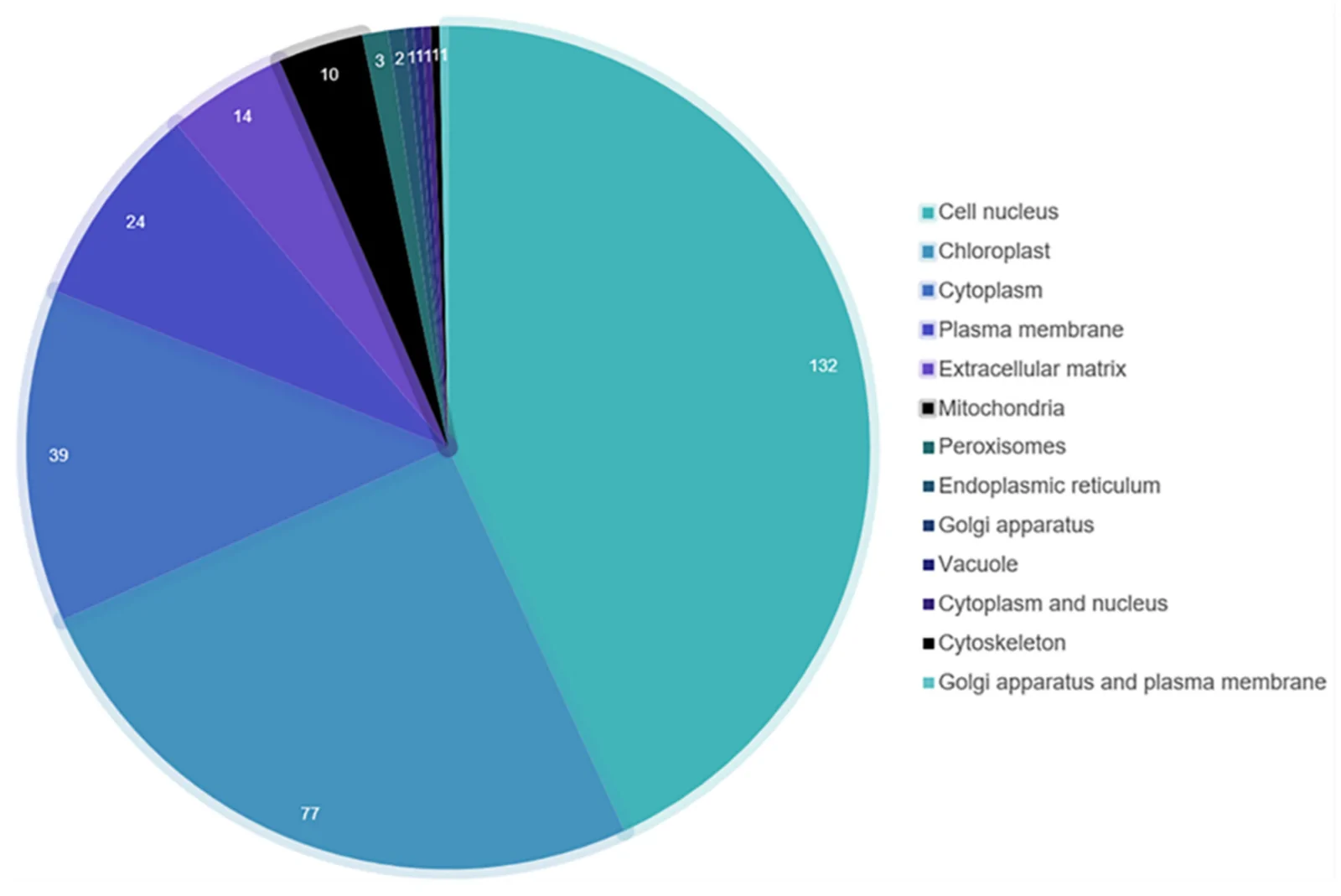
Subcellular localization results of 306 *StRING-zf* genes (primary screening). Colored blocks on the right denote different organelles and the numbers in the figure indicate the number of StRING-zf proteins predicted to be localized in each organelle.

**Figure 2:**
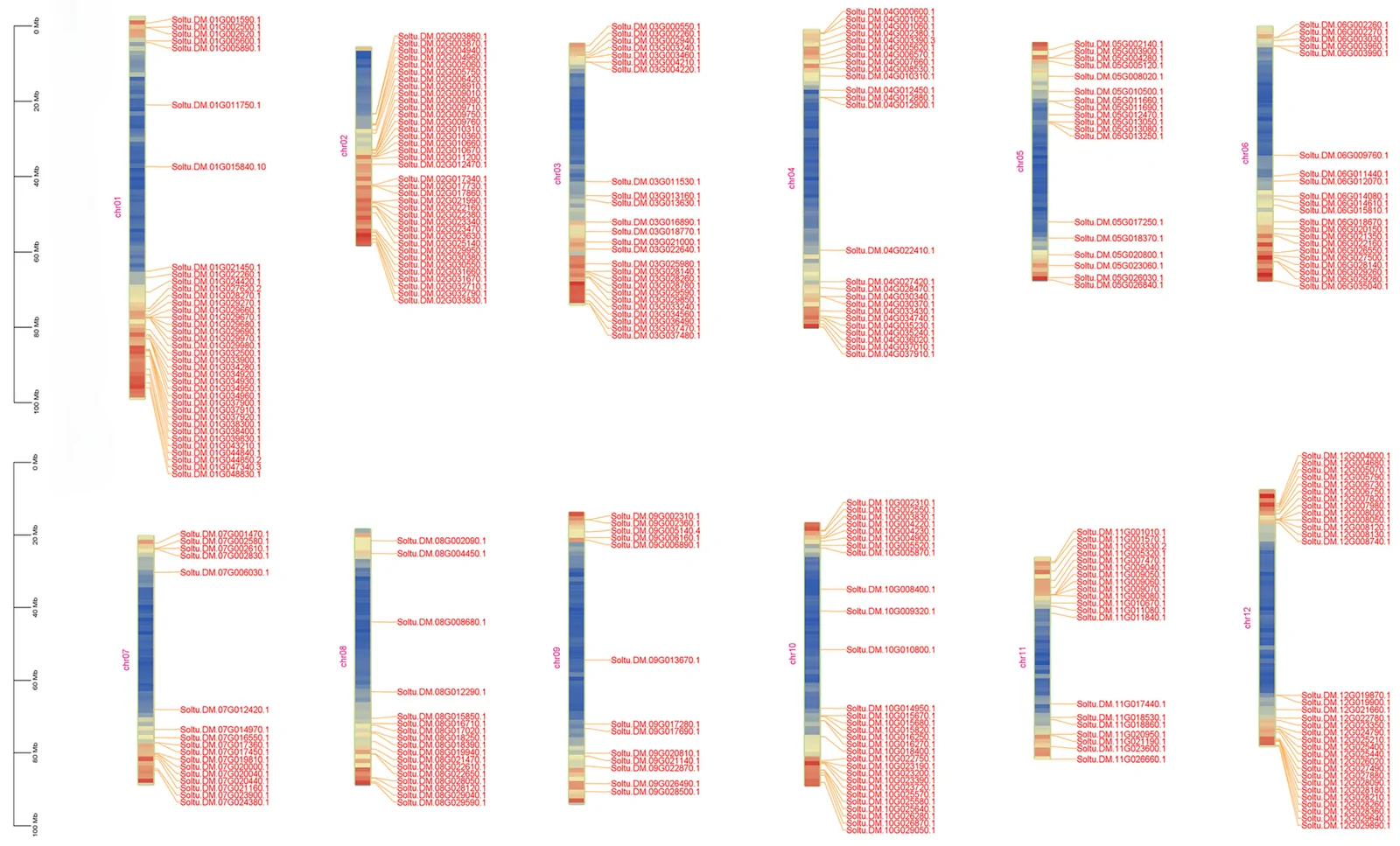
Chromosomal localization of 291 *StRING-zf* genes in potato. The scale on the left represents the length of the chromosome. Chr: Chromosome.

**Figure 3:**
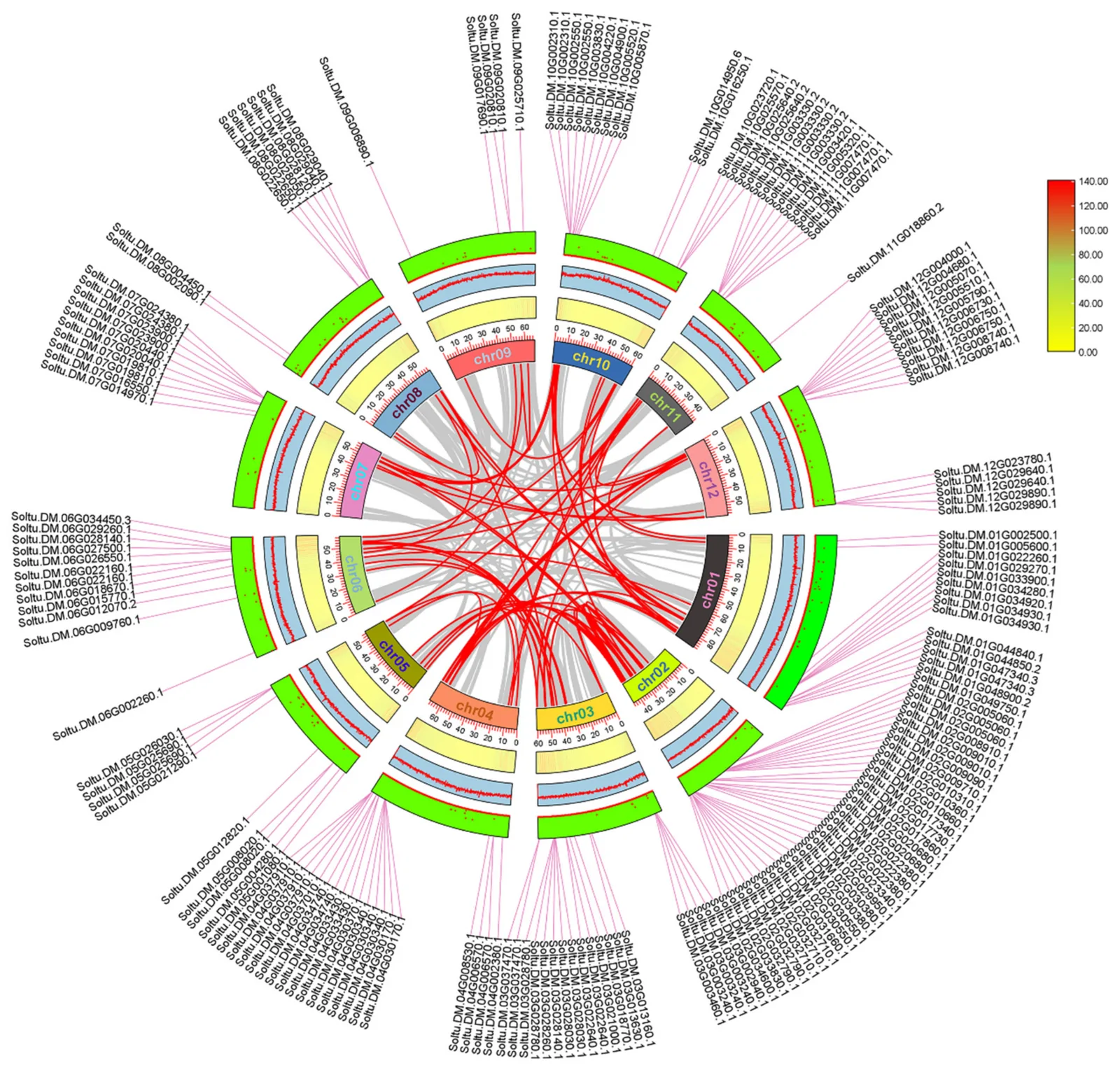
Chromosomal distribution and collinearity analysis of *StRING-zf* genes in potato. Purple lines represent collinear segments within the potato genome connecting *StRING-zf* genes, while red lines indicate duplicated *StRING-zf* genes pairs. Chromosome numbers are shown below each chromosome. Gene density was visualized by hierarchical clustering, with warmer colors indicating higher density and cooler colors indicating lower density. GC content is displayed using line and dot plots, represented by red lines and red dots, respectively.

**Figure 4:**
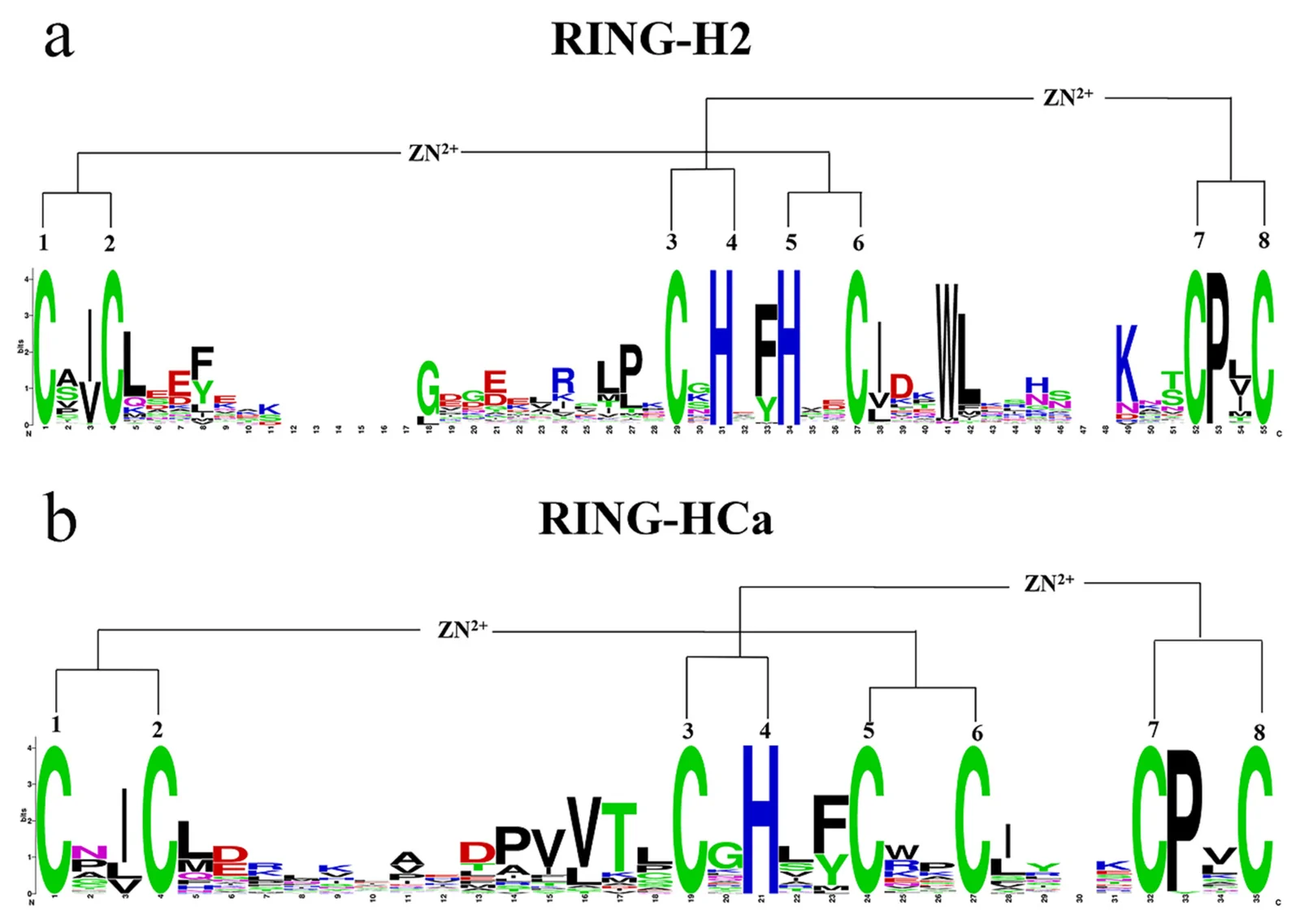
Conserved motif characteristics of the *StRING-zf* genes. The x-axis indicates the positional conservation of the sequence, while the y-axis represents the relative frequency of amino acids at each position within the StRING-zf motif. Sequence logos were generated using the MEME server. (**a**) RING-H2 type; (**b**) RING-HCa type.

**Figure 5:**
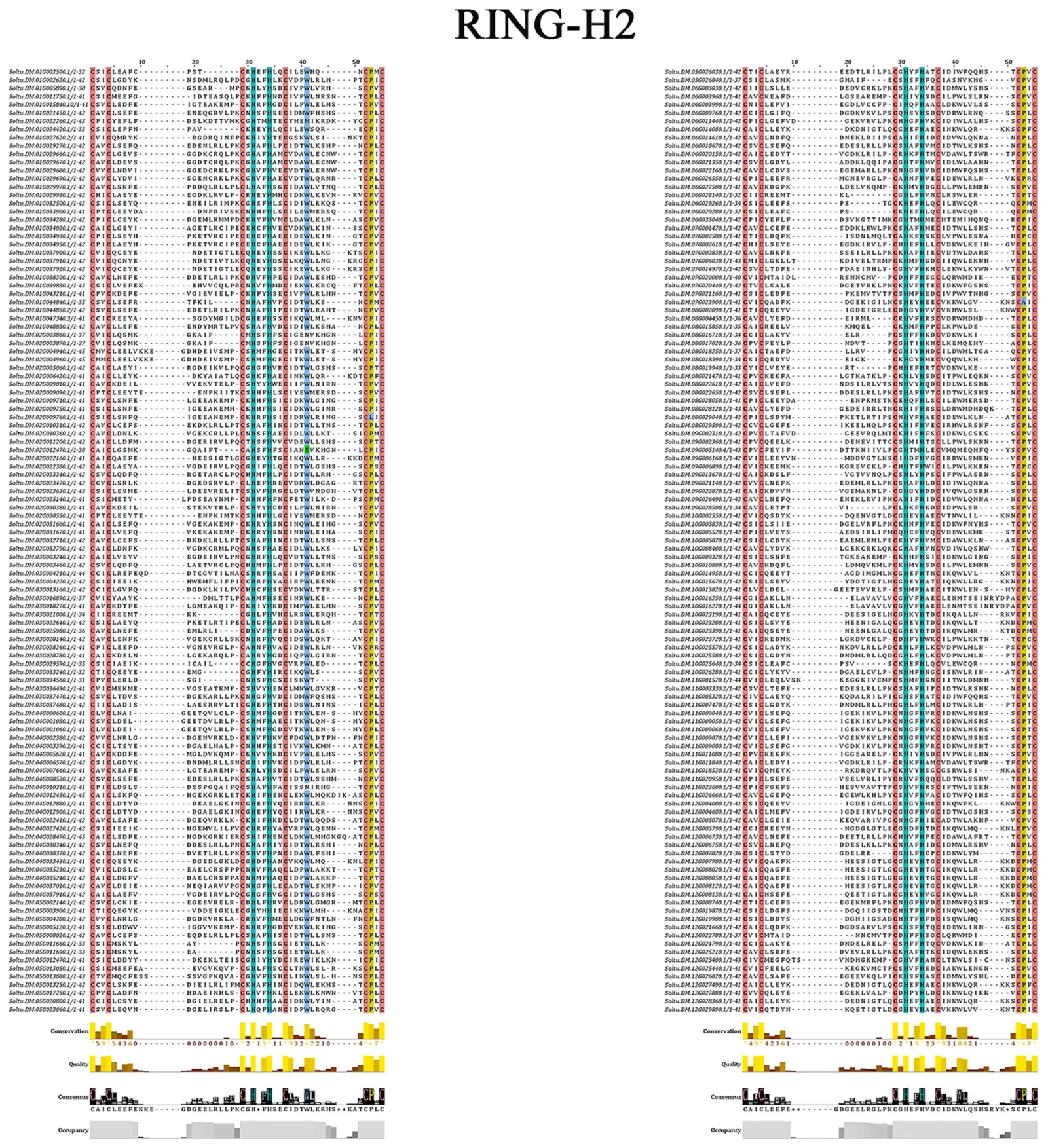
Classification of *StRING-zf* genes. MEME software predicted the conserved functional sequences and genes of StRING-H2.

**Figure 6:**
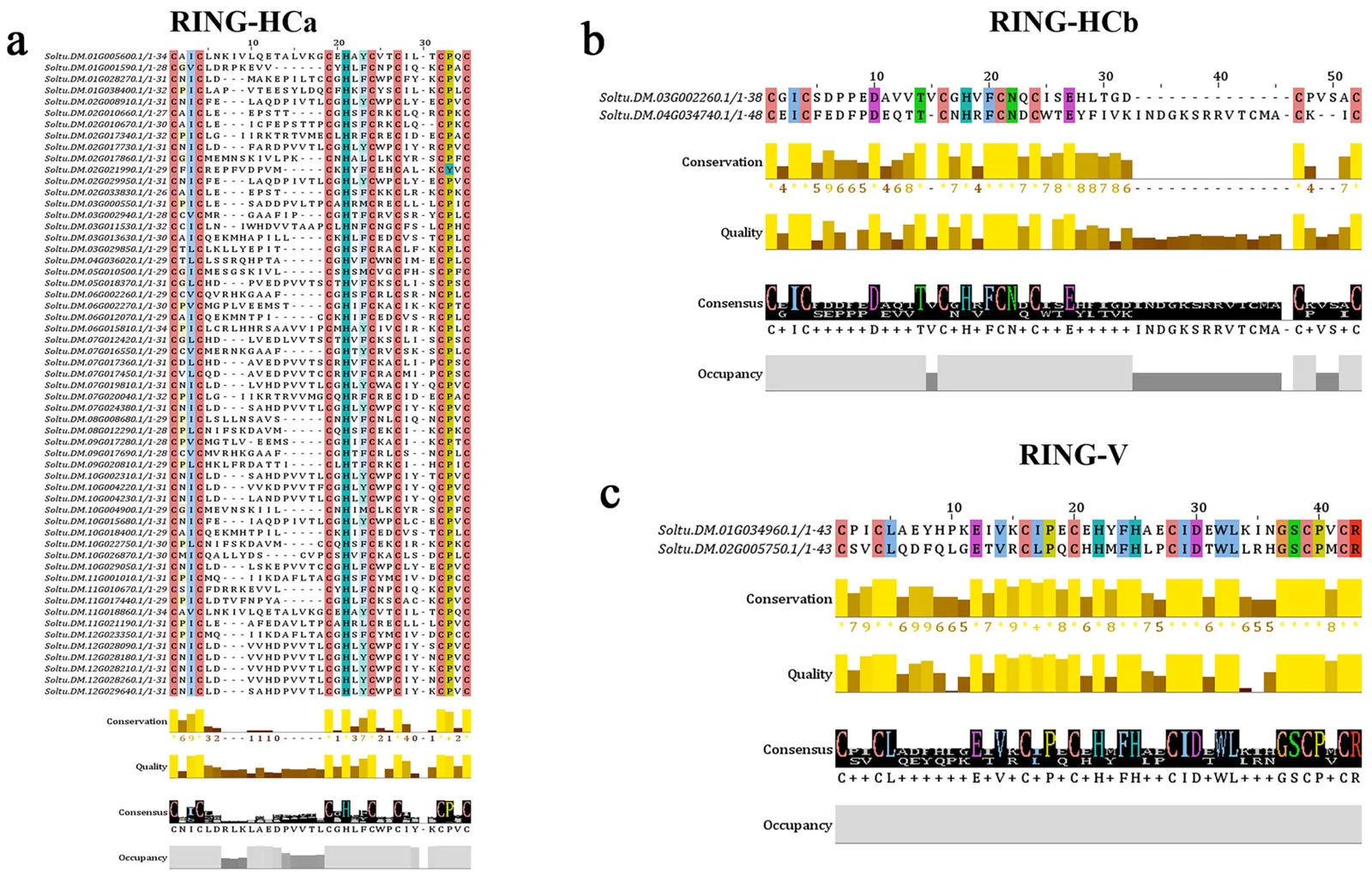
Classification of *StRING-zf* genes. Conserved motifs in the StRING-zf proteins were predicted using the MEME suite. (**a**) StRING-HCa subgroup; (**b**) StRING-HCb subgroup; (**c**) StRING-V subgroup.

**Figure 7:**
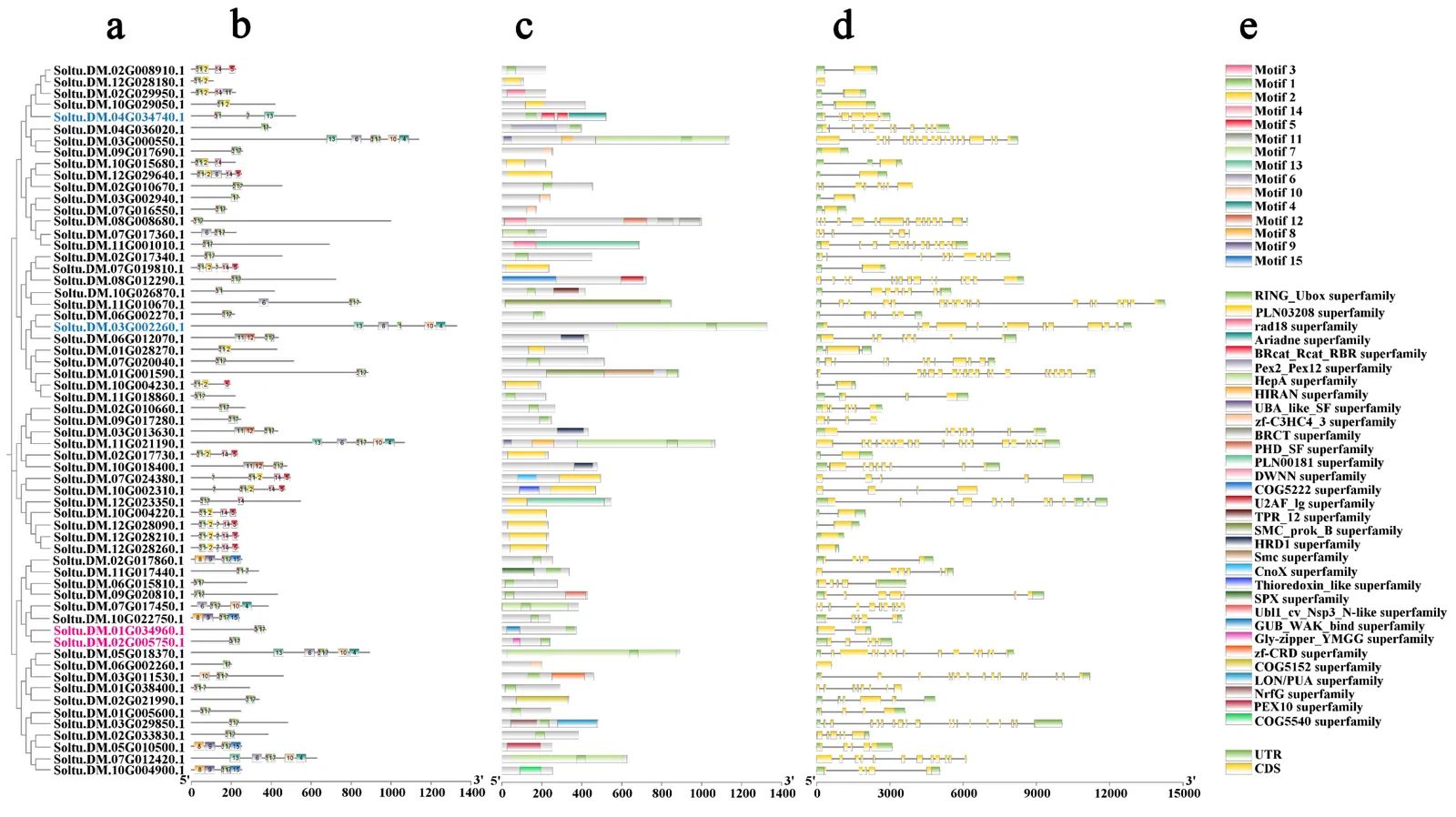
Evolutionary relationships and conserved motifs of StRING-HCa, StRING-HCb, and StRING-V type genes. (**a**) A phylogenetic tree built from the full-length sequences of StRING-zf proteins, with black representing the RING-HCa subgroup, blue for the RING-HCb subgroup, and purple for the RING-V subgroup. (**b**) Structures of conserved motifs in proteins from the three StRING-zf subgroups. The scale at the bottom can be used to estimate the full gene and protein length (unit: aa). (**c**) Types of conserved domains in the proteins of the three StRING-zf subgroups, with the bottom scale for estimating gene and protein lengths (unit: bp). (**d**) Exon, intron, and UTR structures of the three StRING-zf subgroups (unit: bp). Yellow boxes indicate 5′ and 3′ untranslated regions; green boxes indicate exons, and black lines indicate introns. The bottom scale can be used to estimate the full length of genes and proteins. (**e**) A schematic of 15 conserved motifs, conserved protein domains, and gene structures in the three StRING-zf subgroups.

**Figure 8:**
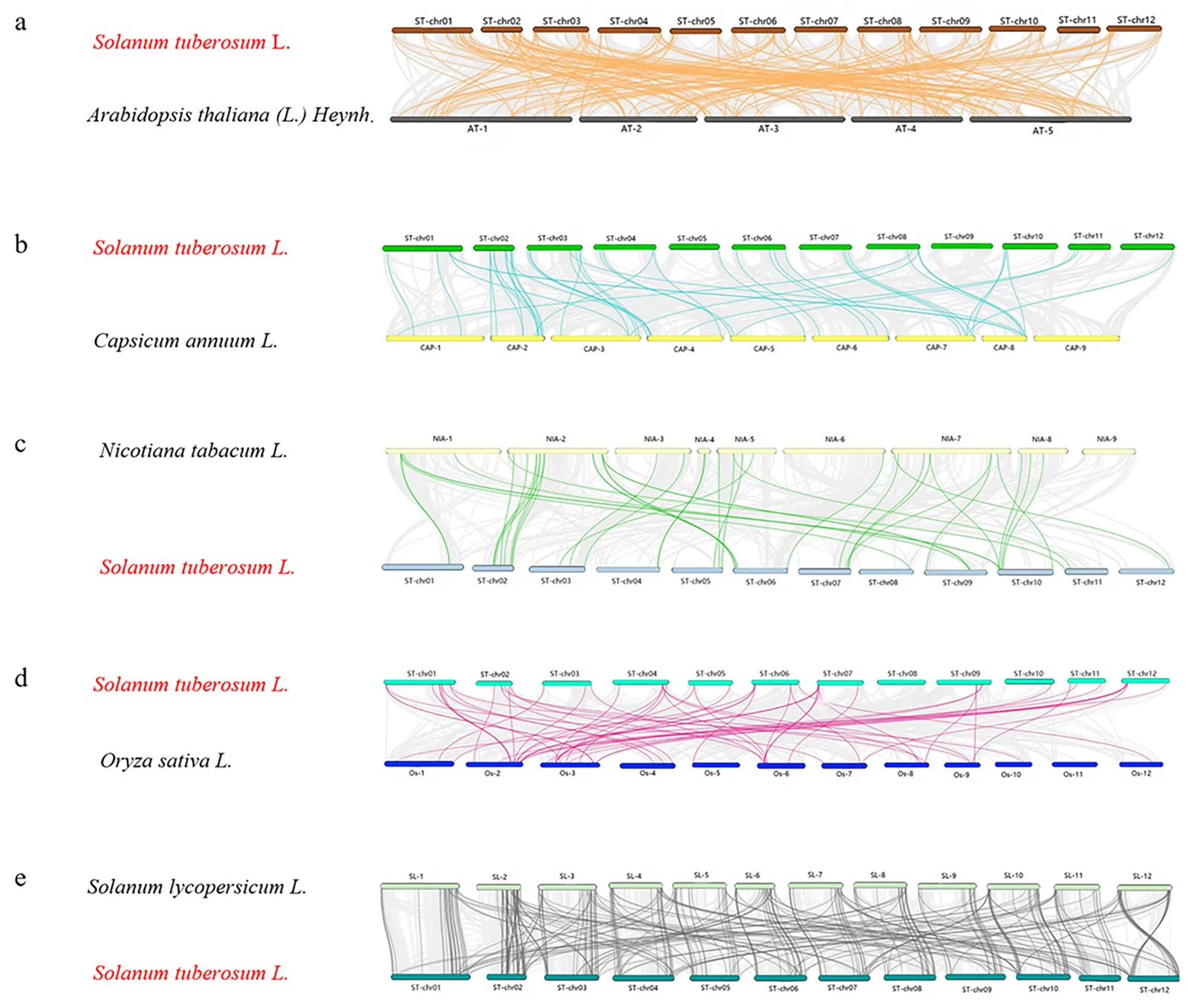
Synteny analysis of *StRING-zf* genes among multiple species. (**a**) Analysis of collinearity between *StRING-zf* genes and Arabidopsis thaliana. (**b**) Collinearity analysis of *StRING-zf* genes and pepper. (**c**) Collinearity analysis of *StRING-zf* genes and tobacco. (**d**) Analysis of collinearity between *StRING-zf* genes and rice. (**e**) Analysis of collinearity between *StRING-zf* genes and tomato. The black font represents the collinear fragments in the whole genomes of the five species, and the red font represents the collinear gene pairs of *StRING-zf* genes.

**Figure 9:**
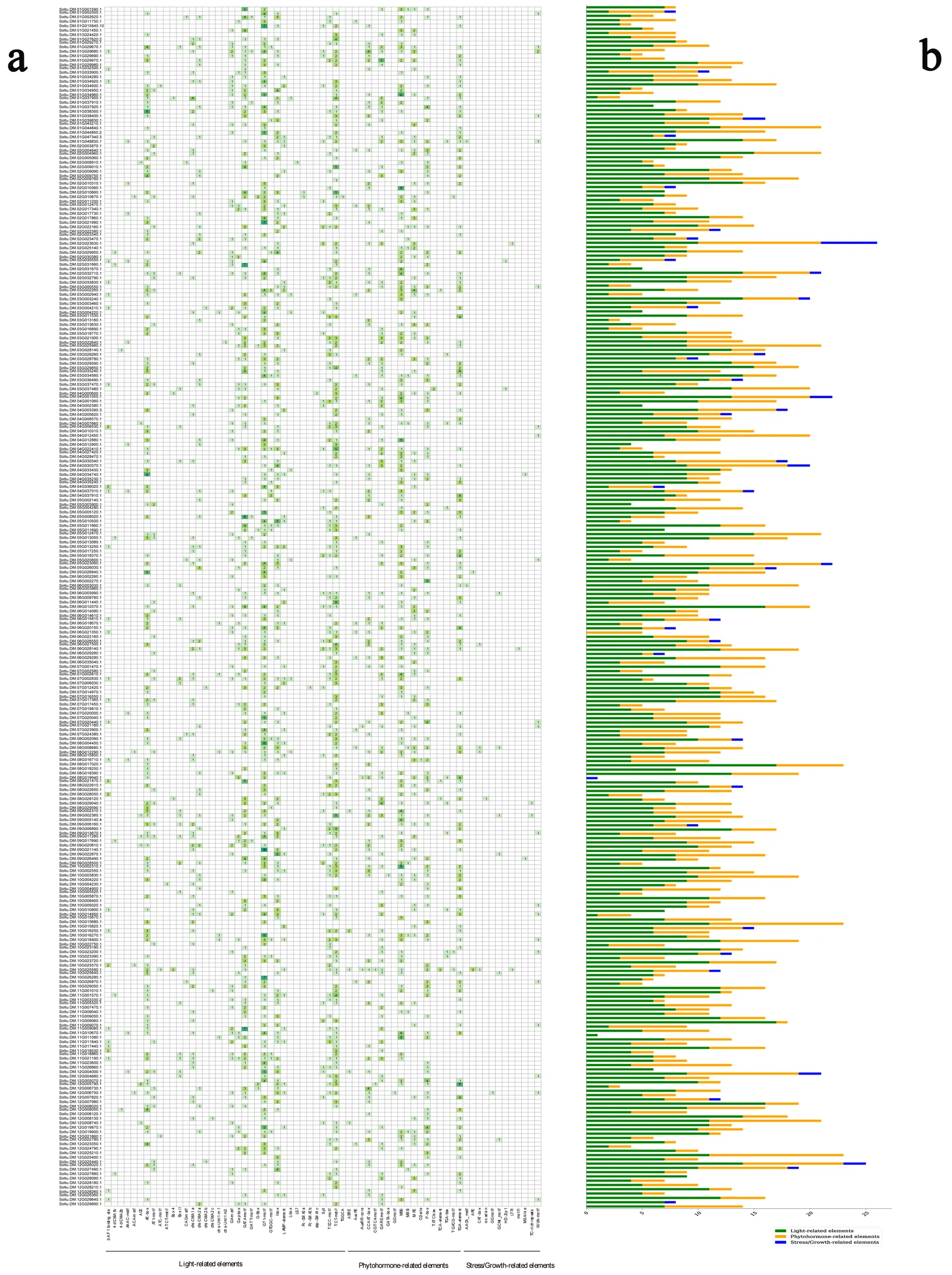
The cis-acting element information of *StRING-zf* genes promoter. (**a**) The cis-acting element of *StRING-zf* genes promoter. (**b**) Quantitative analysis of cis-acting regulatory elements associated with light stress, hormone and abiotic stress response in the promoters of *StRING-zf* genes.

**Figure 10:**
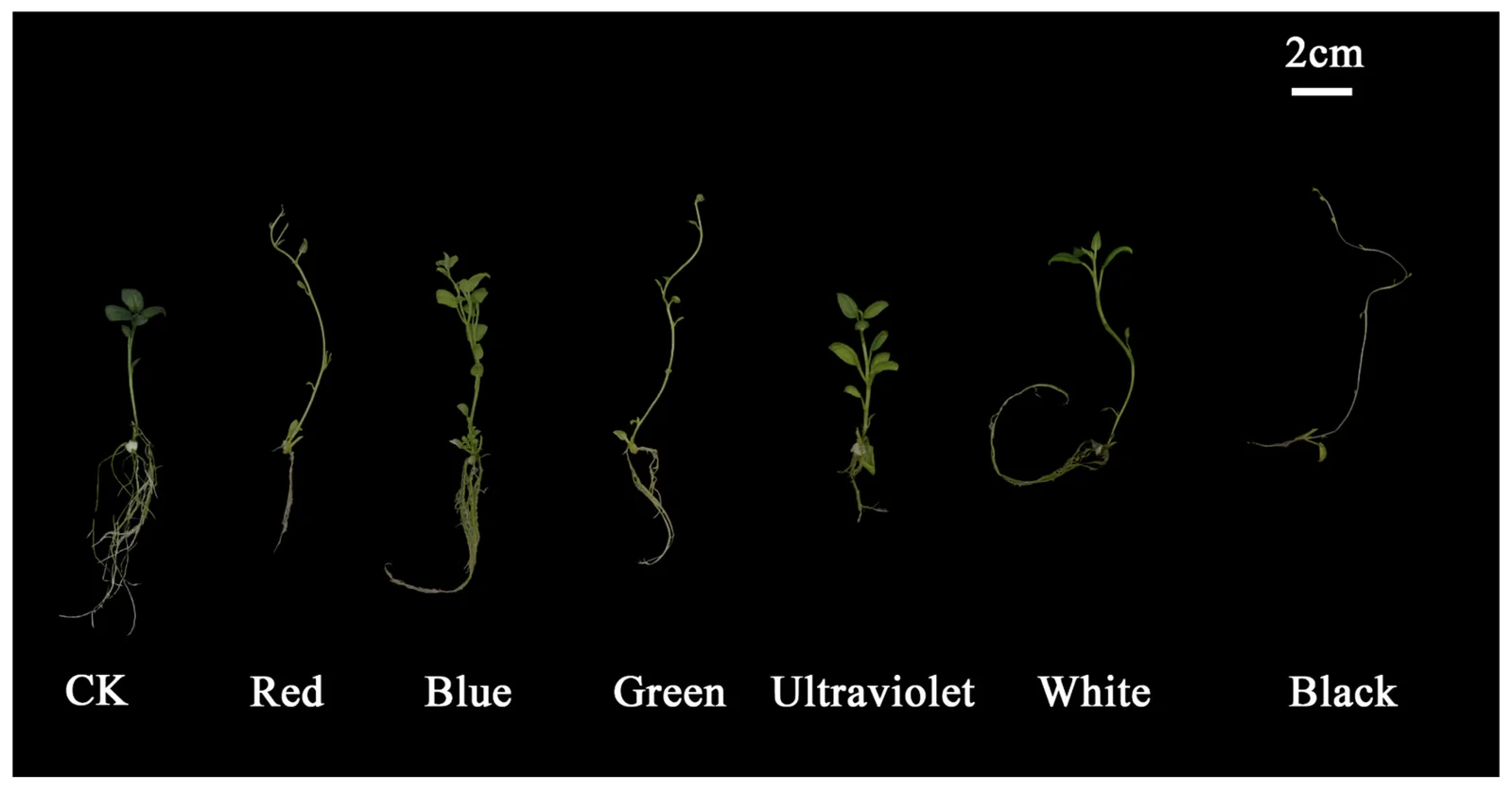
Growth morphology of potato tissue culture seedlings after 28 days under different light qualities.

**Figure 11:**
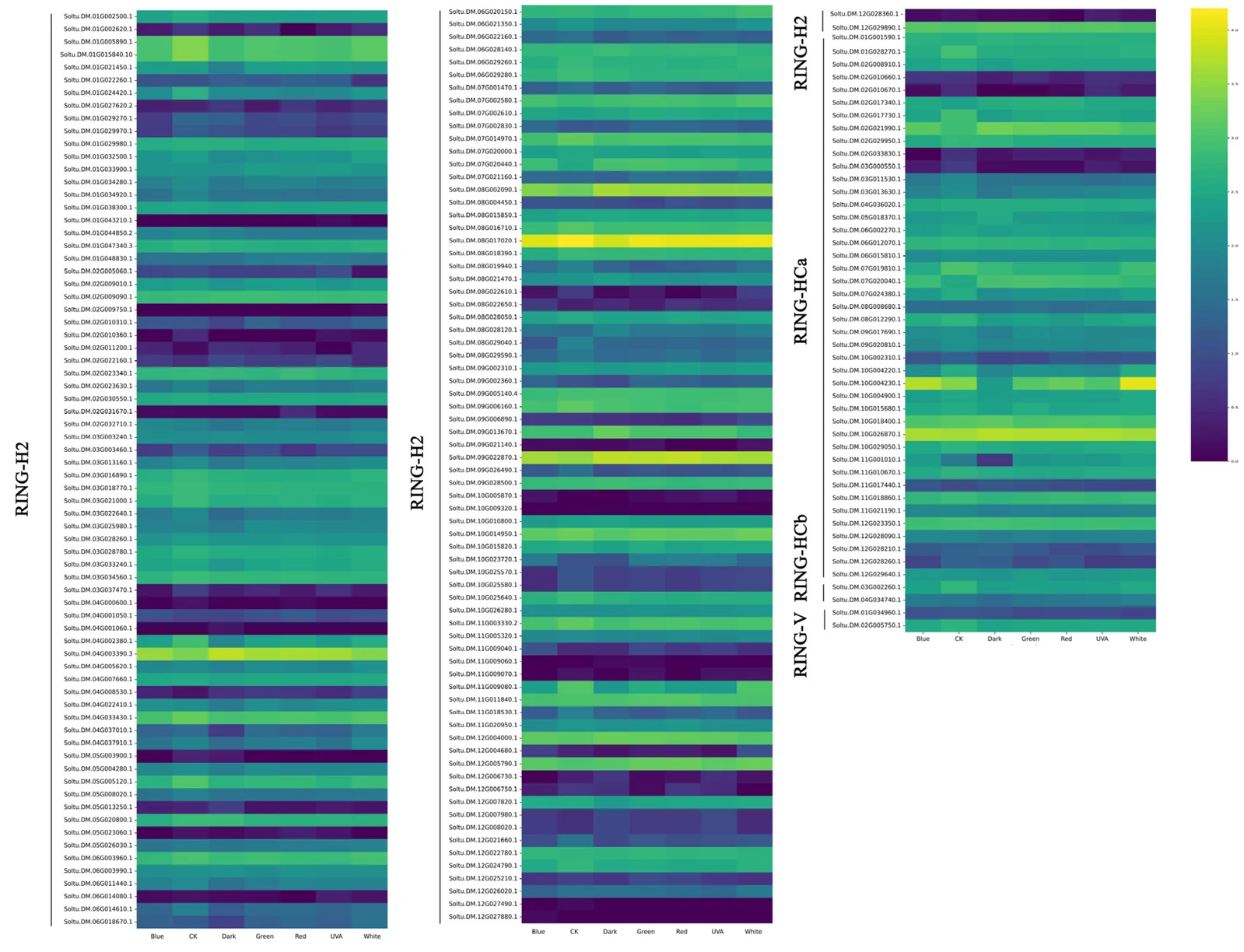
Transcriptome analysis of *StRING-zf* genes. The expression profiles of 193 *StRING-zf* genes under different light treatments. Different treatment types were as follows: blue light (blue), control (CK), dark (dark), green light (green), red light (red), ultraviolet light (UVA), and white light (white). The relative transcriptional abundance spectrum of the gene was hierarchically clustered, and the warmer the color, the higher the expression level. On the right side, the names and subfamily types of each gene are marked.

**Figure 12:**
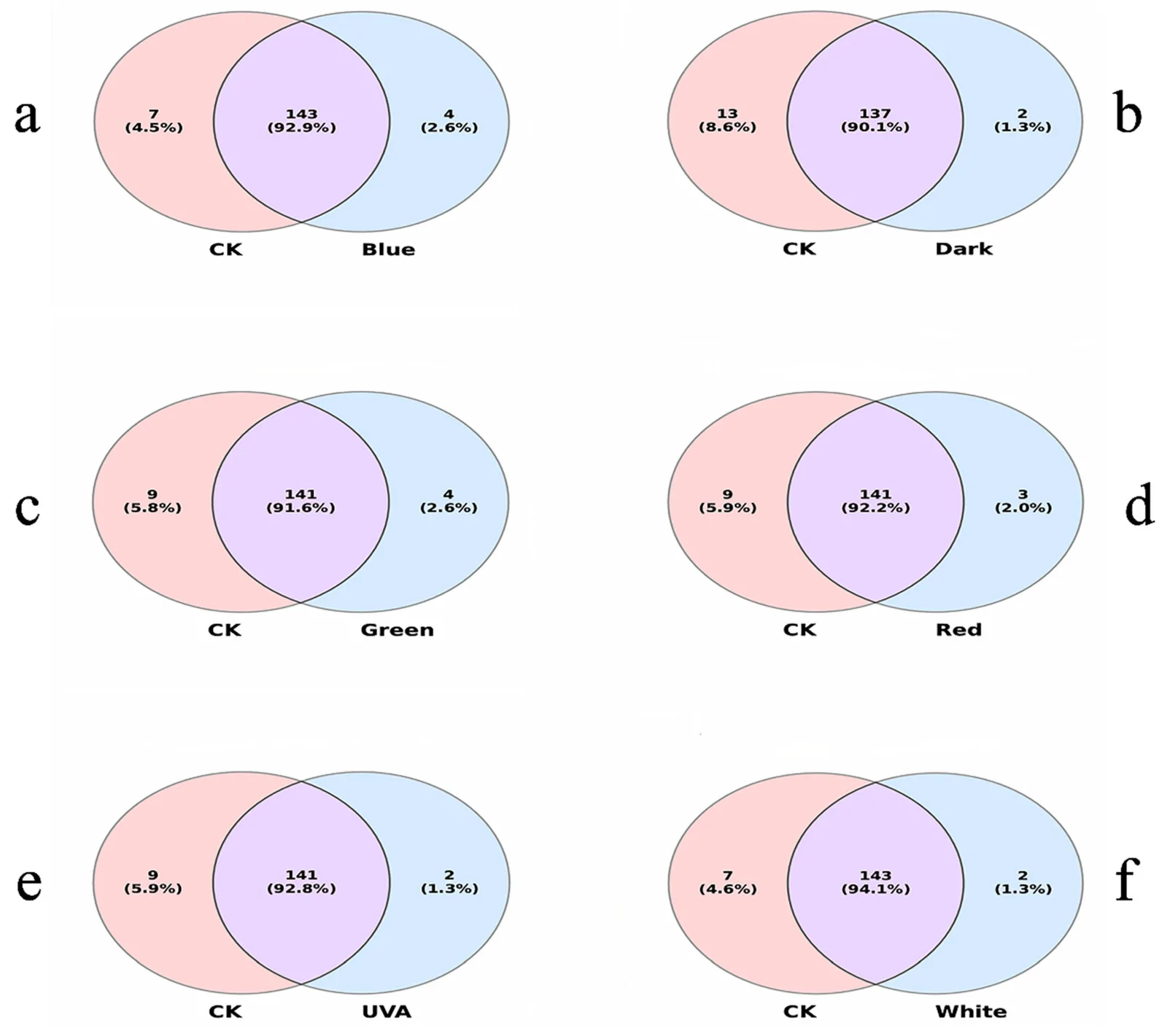
Venn diagram between different light treatments. (**a**) Gene intersection of CK and blue. (**b**) Gene intersection of CK and dark. (**c**) Gene intersection of CK and green. (**d**) The gene intersection of CK and red. (**e**) The gene intersection of CK and UVA. (**f**) The gene intersection of CK and white.

**Figure 13:**
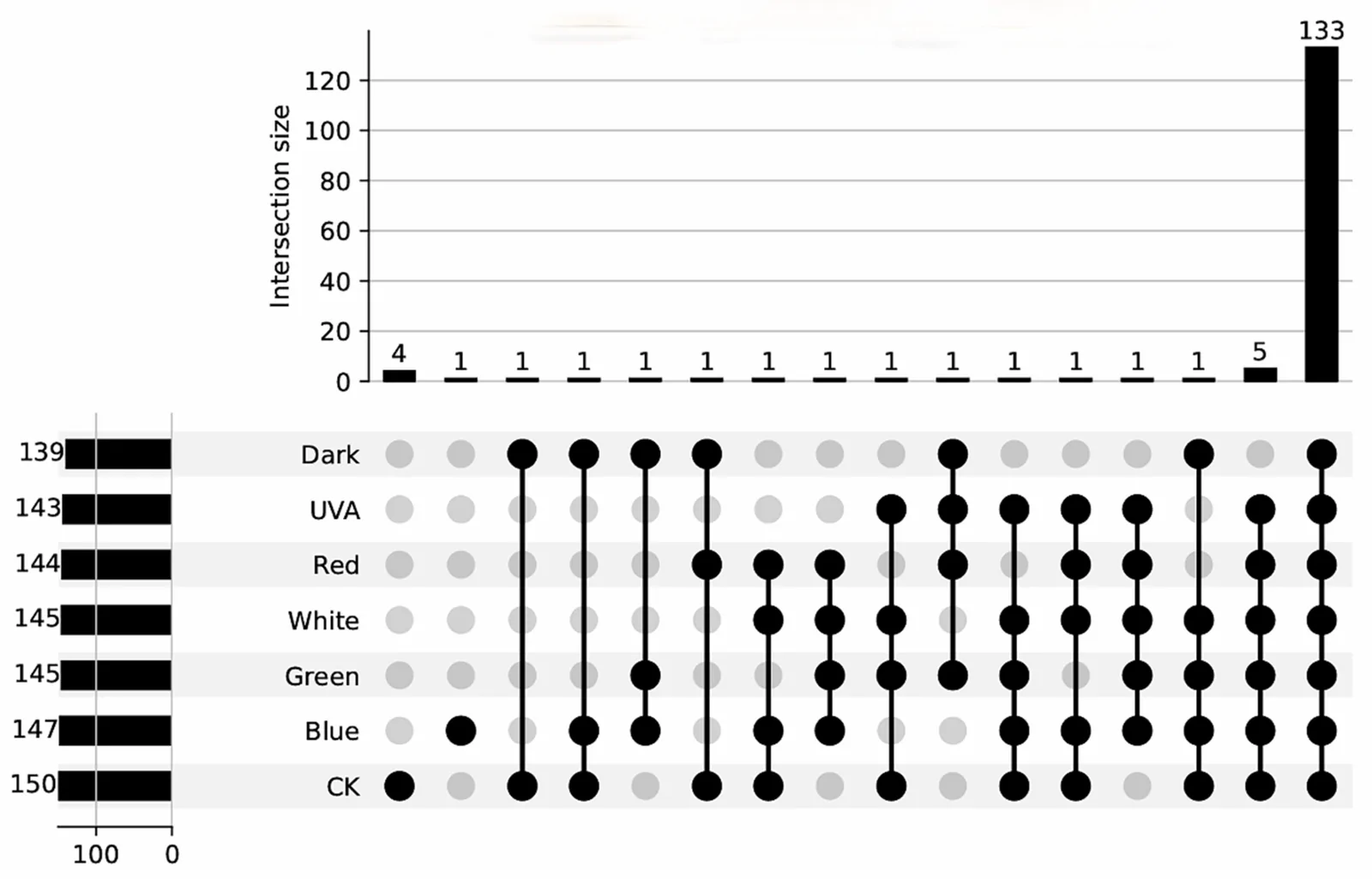
Upset diagram of the intersection relationship of multiple sets of gene expression data. Diagram showing the number of genes with increased expression levels under different types of light treatment. The top histogram shows the number of genes highly expressed in one or more treatments (Y-axis); the corresponding combinations of light variants (X-axis) are indicated by filled circles in the underlying histogram. The bottom histogram also shows the number of highly expressed genes in each light treatment (on the left on the y-axis). This number is the sum of the number of genes in the upper histogram corresponding to the filled circles in the lower histogram.

**Figure 14:**
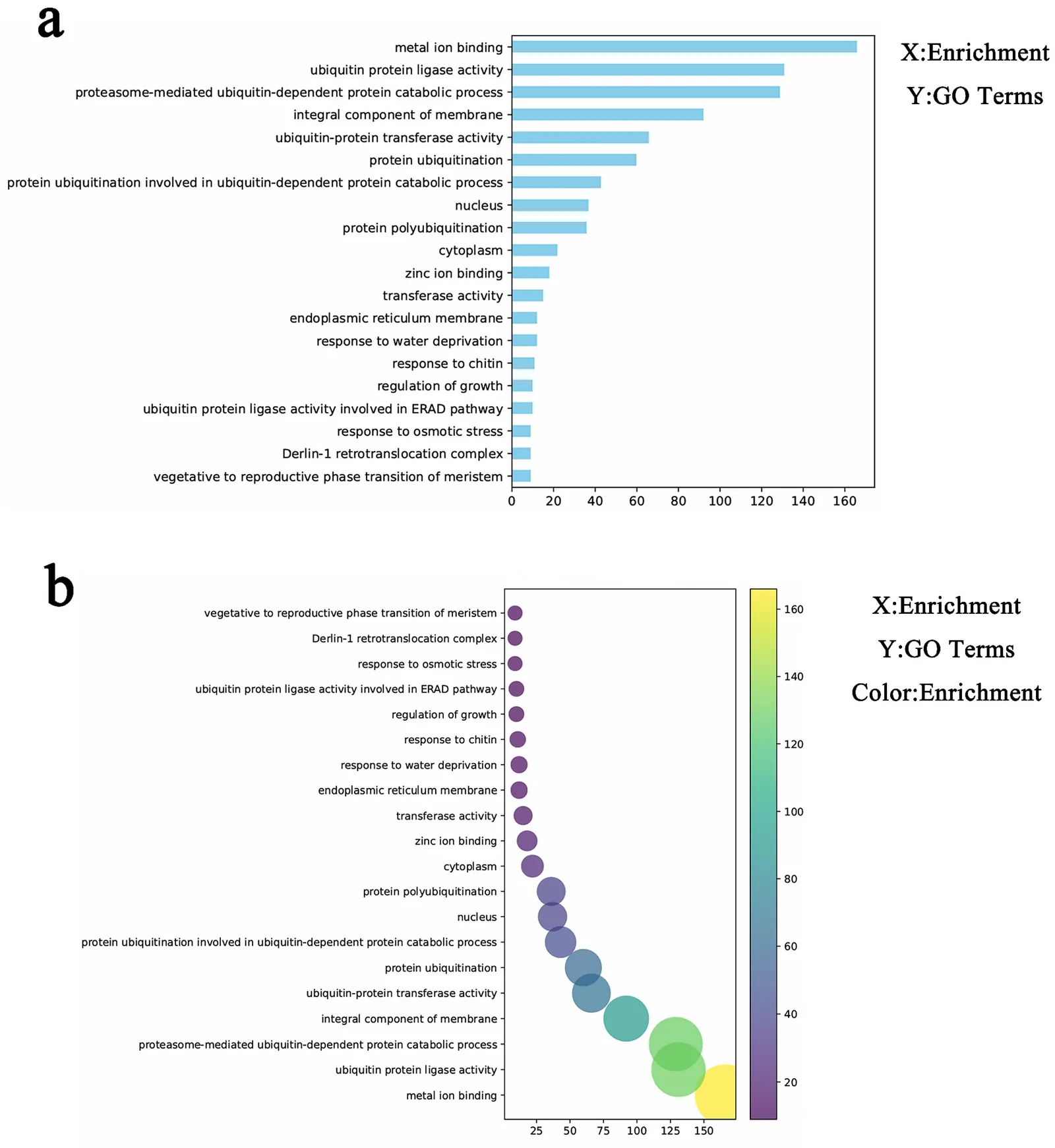
Functional annotation and enrichment analysis of *StRING-zf* genes. The GO annotations of 193 *StRING-zf* genes were counted, and high-frequency GO terms were screened. (**a**) Bar plot of enriched GO terms, with the x-axis indicating enrichment level and the y-axis indicating GO terms. (**b**) Bubble plot of enriched GO terms, with the x-axis indicating enrichment level and the y-axis indicating GO terms. Bubble size represents enrichment level, and the color gradient from purple to yellow indicates increasing enrichment.

**Figure 15:**
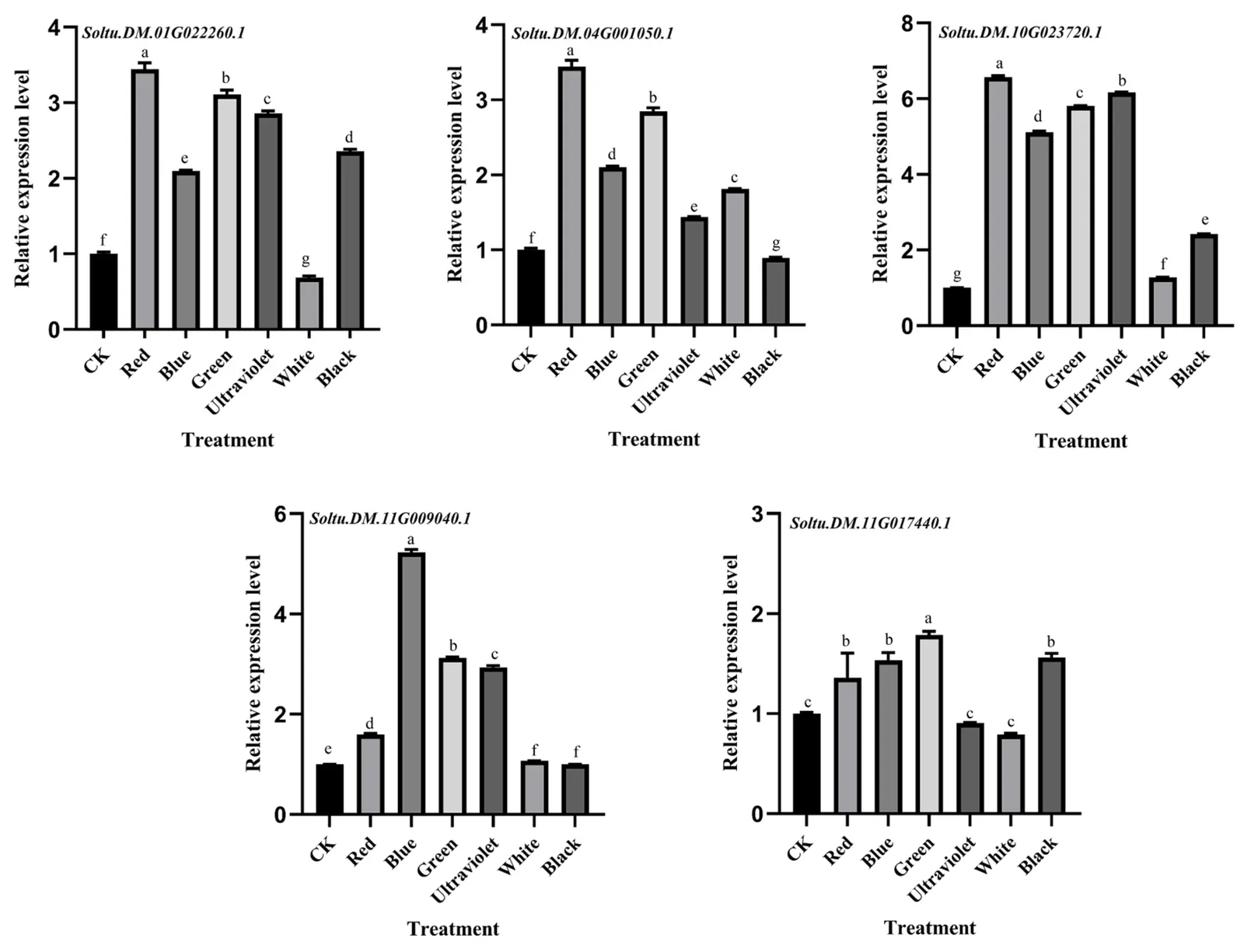
Quantitative RT-PCR analysis of 5 candidate *StRING-zf* genes. The expression of five candidate genes under different light qualities. Different lowercase letters above the bars indicate significant differences among treatments within the same gene (*p* < 0.05), whereas bars sharing the same letter are not significantly different.

**Table 1:** Types and properties of RING domains in potato.

RING Domain	Consensus Sequence															
Type	No.	ml1		ml2		ml3		ml4		ml5		ml6		ml7		ml8
RING-H2	230	C	X_2_	C	X_11–33_	C	X_1_	H	X_2,3_	H	X_2_	C	X_7–64_	C	X_2_	C
RING-Hca	57	C	X_2_	C	X_10–33_	C	X_1–3_	H	X_2,3_	C	X_2_	C	X_6–30_	C	X_2_	C
RING-Hcb	2	C	X_2_	C	X_11–18_	C	X_1,2_	H	X_2_	C	X_2_	C	X_7–19_	C	X_3,4_	C
RING-v	2	C	X_2_	C	X_8–29_	C	X_1–4_	C	X_4–7,9_	H	X_2_	C	X_9–36_	C	X_2_	C

**Note:** Eight conserved metal ligands (MLs) are listed in the table. X(n) indicates the number of amino acids between two conserved metal ligands, and “No.” represents the number of genes in each RING domain type.

**Table 2:** Ka/Ks of *Solanum tuberosum* L. with four dicotyledons.

Dicotyledon	Ka/Ks
*Arabidopsis thaliana*	0.19
*Solanum lycopersicum*	0.30
*Capsicum annuum* L.	0.28
*Nicotiana tabacum*	0.27

**Note:** The Ka/Ks ratio is an indicator to measure the evolutionary selection pressure of gene.

**Table 3:** Effects of different light qualities on physiological characteristics of potato tissue culture seedlings (plant height, stem diameter, leaf number, maximum leaf area).

Treatment	Plant Height (cm)	Stem Diameter (mm)	Number of Leaves	Maximum Leaf Area (cm^2^)
CK	9.22 ± 0.69 c	1.15 ± 0.09 c	13 ± 1.28 c	1.16 ± 0.04 a
Red	10.06 ± 0.78 b	0.77 ± 0.08 d	12 ± 1.18 cd	0.16 ± 0.02 f
Blue	8.66 ± 0.71 c	1.78 ± 0.39 a	16 ± 2.02 a	0.75 ± 0.08 d
Green	14.55 ± 1.00 a	0.64 ± 0.07 e	11 ± 2.02 d	0.35 ± 0.02 e
Ultraviolet	5.13 ± 0.44 d	1.08 ± 0.18 c	12 ± 2.09 cd	0.99 ± 0.05 c
White	10.35 ± 0.83 b	1.27 ± 0.07 b	15 ± 1.32 b	1.08 ± 0.09 b
Black	14.71 ± 1.28 a	0.51 ± 0.09 f	10 ± 0.94 e	0.04 ± 0.01 g

**Note:** The data in the table are displayed as follows: average ± standard deviation; different lowercase letters indicate significant differences at *p* < 0.05.

**Table 4:** Effects of different light qualities on the physiological characteristics of potato tissue culture seedlings (the longest root length, root number, fresh weight per plant and dry weight per plant).

Treatment	Longest Root Length (cm)	Number of Roots	Fresh Weight per Plant (g)	Total Dry Weight (g)
CK	9.34 ± 0.48 b	9 ± 0.72 a	0.70 ± 0.10 a	0.14 ± 0.01 b
Red	9.89 ± 1.12 a	7 ± 1.21 b	0.42 ± 0.07 c	0.14 ± 0.02 b
Blue	8.59 ± 0.50 c	6 ± 1.13 c	0.37 ± 0.02 de	0.12 ± 0.01 c
Green	8.57 ± 0.98 c	4 ± 2.09 d	0.35 ± 0.02 e	0.10 ± 0.02 d
Ultraviolet	6.74 ± 0.38 e	8 ± 0.86 b	0.64 ± 0.05 b	0.08 ± 0.01 e
White	7.87 ± 0.58 d	6 ± 1.22 c	0.66 ± 0.04 ab	0.16 ± 0.01 a
Black	3.14 ± 0.46 f	4 ± 0.88 d	0.40 ± 0.05 cd	0.07 ± 0.01 e

**Note:** The data in the table are displayed as follows: average ± standard deviation; different lowercase letters indicate significant differences at *p* < 0.05.

## Data Availability

The original data presented in the study are included in the article and Supplementary Information; further inquiries can be directed to the corresponding authors The data that support the findings of this study are available from the corresponding authors upon reasonable request due to privacy.

## Supplementary Materials

The following supporting information can be downloaded at https://www.mdpi.com/article/10.3390/plants15192915/s1, Supplementary File S1: Tables S1–S3 (RING domain genes identified); Supplementary File S2: Tables S1–S6 (relationships within and between species); Supplementary File S3: Tables S1 and S2 (cis-acting element); Supplementary File S4: Tables S1 and S2 (expression values); Supplementary File S5: Table S1 (Venn CK vs other genes); Supplementary File S6: Table S1 (GO number, content, and count summary); Supplementary File S7: Table S1 (the list of primers); Supplementary Figure S1 (evolutionary relationships and conserved motifs of the StRING-H2 genes).
