# Supplementary material for: Genome-Wide Characterization and Transcriptomic Analysis Reveal Light-Quality-Responsive RING Zinc Finger Genes in Potatoes
Source: Plants (Basel). 2026 Sep 23;15(19):2915. doi: 10.3390/plants15192915 (PMC13667170; doi:10.3390/plants15192915)
Supplement: Supplementary file 1 [file plants-15-02915-s001.zip › Supplementary Figure S1.pdf]

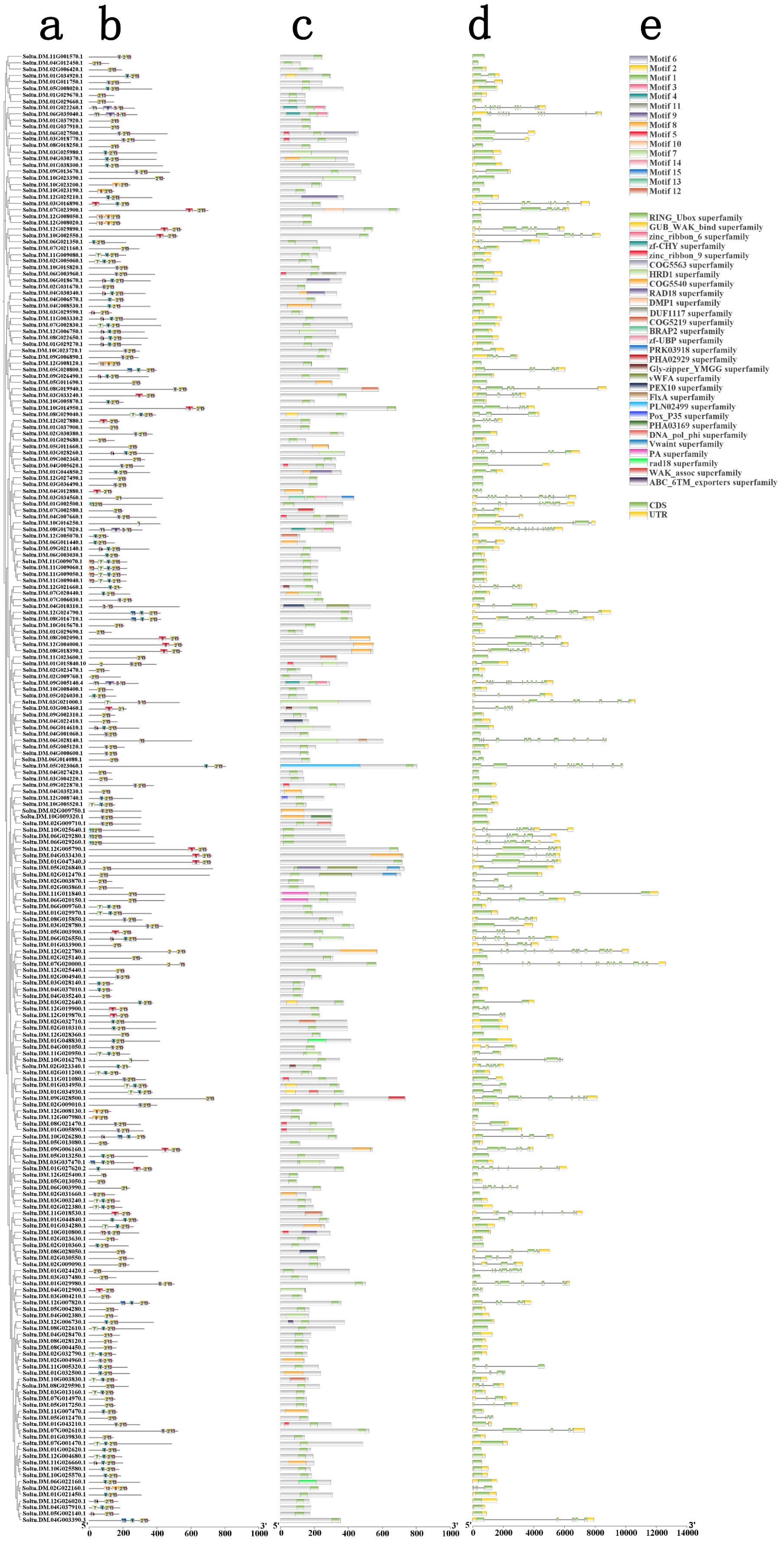

a
b
c
d
e
Motif 6
Motif 2
Motif 1
Motif 3
Motif 4
Motif 11
Motif 9
Motif 8
Motif 5
Motif 10
Motif 7
Motif 14
Motif 15
Motif 13
Motif 12
RING_Ubox superfamily
GUB_WAK_bind superfamily
zinc_ribbon_6 superfamily
zf-CHY superfamily
zinc_ribbon_9 superfamily
COG5563 superfamily
HRD1 superfamily
COG5540 superfamily
RAD18 superfamily
DMP1 superfamily
DUF1117 superfamily
COG5219 superfamily
BRAP2 superfamily
zf-UBP superfamily
PRK03918 superfamily
PHA02929 superfamily
Gly-zipper_YMGG superfamily
vWFA superfamily
PEX10 superfamily
FlxA superfamily
PLN02499 superfamily
Pox_P35 superfamily
PHA03169 superfamily
DNA_pol_phi superfamily
Vwaint superfamily
PA superfamily
rad18 superfamily
WAK_assoc superfamily
ABC_6TM_exporters superfamily
CDS
UTR
5'
3'
0
200
400
600
800
1000
2000
4000
6000
8000
10000
12000
14000

Figure S1 Evolutionary relationships and conserved motifs of the *StRING-H2* genes. a. A phylogenetic tree of full-length StRING-H2 protein sequences. b. Structures of conserved motifs in StRING-H2 proteins; the scale at the bottom can be used to estimate the full length of the gene and protein(Unit: aa). c. Types of conserved domains in StRING-H2 proteins; the scale at the bottom can be used to estimate the full length of the gene and protein(Unit: bp). d. Exon, intron, and UTR structures of *StRING-H2*. Yellow boxes indicate 5' and 3' untranslated regions, green boxes indicate exons, and black lines indicate introns(Unit: bp). The scale at the bottom can be used to estimate the full length of the gene and protein. e. Diagram showing 15 conserved motifs（b）, protein conserved domains(c), and gene structures of *StRING-H2*(d).
